# New generation of hydraulic pedotransfer functions for Europe

**DOI:** 10.1111/ejss.12192

**Published:** 2014-11-03

**Authors:** B Tóth, M Weynants, A Nemes, A Makó, G Bilas, G Tóth

**Affiliations:** a University of Pannonia, Georgikon Faculty, Department of Crop Production and Soil ScienceDeák F. u. 16, Keszthely, 8360, Hungary; b European Commission, Joint Research Centre (JRC), Institute for Environment and Sustainability (IES), Land Resource Management Unit, Via Enrico Fermi 274921027 Ispra VA, Italy; c Bioforsk, Soil and EnvironmentFrederik A. Dahls vei 20, Ås, 1430, Norway; d Hungarian Academy of Sciences, Centre for Agricultural Research, Institute for Soil Sciences and Agricultural ChemistryHerman Ottó út 15, Budapest, 1022, Hungary; e Aristotle University of Thessaloniki, School of Agriculture, Lab of Applied Soil Science, University Campus, UB 25954124, Thessaloniki, Greece

## Abstract

A range of continental-scale soil datasets exists in Europe with different spatial representation and based on different principles. We developed comprehensive pedotransfer functions (PTFs) for applications principally on spatial datasets with continental coverage. The PTF development included the prediction of soil water retention at various matric potentials and prediction of parameters to characterize soil moisture retention and the hydraulic conductivity curve (MRC and HCC) of European soils. We developed PTFs with a hierarchical approach, determined by the input requirements. The PTFs were derived by using three statistical methods: (i) linear regression where there were quantitative input variables, (ii) a regression tree for qualitative, quantitative and mixed types of information and (iii) mean statistics of developer-defined soil groups (class PTF) when only qualitative input parameters were available. Data of the recently established European Hydropedological Data Inventory (EU-HYDI), which holds the most comprehensive geographical and thematic coverage of hydro-pedological data in Europe, were used to train and test the PTFs. The applied modelling techniques and the EU-HYDI allowed the development of hydraulic PTFs that are more reliable and applicable for a greater variety of input parameters than those previously available for Europe. Therefore the new set of PTFs offers tailored advanced tools for a wide range of applications in the continent.

## Introduction

Numerous pedotransfer functions (PTFs) have been developed in Europe in recent decades (Vereecken *et al.*, 1989, 1990; Børgesen & Schaap, 2005; Baker & Ellison, 2008; Weynants *et al.*, 2009). Many of them are very accurate but applicable only to limited areas. These PTFs therefore have limited validity when considered for continental scale applications. Up to now, continuous and class PTFs developed from the HYPRES data-base (Wösten *et al.*, 1999) are the only ones intended and available to predict soil hydraulic properties for continental scale applications in Europe. However, the HYPRES-based PTFs have a number of limitations. For example, HYPRES holds data mainly from Western European countries and is not representative of Central and Eastern Europe. Other shortcomings include unpublished accuracy figures and the absence of the assessment of the importance of variables for restricting inputs for those which improve predictions. In addition, the HYPRES-based PTFs do not consider chemical properties, which may improve hydraulic predictions in certain cases.

Since the publication of the HYPRES-based PTFs, major developments have occurred in the availability of measured hydro-pedological and general soil survey data, as well as in modelling procedures and tools. Today a wider range of soil properties can be considered for model development (new training datasets) and implementation, as in new digital soil property maps (Adhikari *et al.*, 2013; Arrouays *et al.*, 2014).

Soil hydraulic parameters needed in environmental, hydrological or land-surface modelling vary according to the model used. Some models require knowledge of the whole moisture retention curve (MRC) (MohidLand, SWAP, CLM, HYDRUS). Other models, for example the SWAT model, require water retention values at given matric potentials. Direct point predictions for given matric potentials can lead to more accurate estimations than if water retention values of these matric potentials are derived from predicted MRCs (parameter estimation) (Pachepsky *et al.*, 1996; Børgesen & Schaap, 2005). Therefore it is important to have both point predictions and parameter estimations.

It is also known that the performance of prediction models is highly dependent on the characteristics of the data (number and kind of measured properties, sample size and heterogeneity) used for their development. Nemes *et al.* (2003) underlined the need to assemble a comprehensive dataset containing soil taxonomic, chemical, physical, hydrological and land cover/use data in Europe. New predictions should also consider the underlying characteristics and spatial extent of the information that is readily available or foreseen, such as the European coverage of the upcoming GlobalSoilMap (Arrouays *et al.*, 2014), for spatial applications of the PTFs. The recent construction of the European Hydropedological Data Inventory (EU-HYDI), which gathers contributions from 18 European countries (Weynants *et al.*, 2013), has provided the opportunity to establish new PTFs based on the above principles, including the consideration of all soil characteristics available on continental maps.

The aim of this study is to provide point and parametric PTFs of soil hydraulic properties for applications in Europe with a hierarchical input data approach. A systematic assessment of data available for continental soil hydrological applications was performed. We focused on predictions based on input parameters that are available in continental-scale spatial layers in Europe, thus enabling users to implement the functions at this scale. A series of statistical methods was tested and applied for the development of PTFs. Results of the most reliable methods are presented in a hierarchical structure by the extent of requirement for inputs.

## Materials and methods

This section provides only the essential features of the material and methods used and further details are given in File S1.

### Dataset

PTFs were developed with data from EU-HYDI (Weynants *et al.*, 2013). The EU-HYDI was built as a collective effort of 29 institutes in 18 European countries and contains information on taxonomic, chemical and physical soil properties and data on land use for 18 537 unique soil samples from 6460 soil profiles across the continent. Weynants *et al.* (2013) provide full details of the dataset, including methodology, characteristics of the samples and the data harmonization that took place prior to its release. File S1 describes the preparation and filtering of the data for the current analysis. For the predictions we used variables available in EU-HYDI that are also available in possible implementation datasets (Table1). The order of soil input parameters to be included in models was based on their availability for larger European areas, such as river catchments or geographical regions. It is noted, that application of the PTFs developed is not limited to the databases listed in Table1. The new PTFs can also be applied for other soil data, such as continuous maps or profile information from within Europe, as long as the necessary inputs exist.

**Table 1:** Continental soil datasets in Europe available or foreseen for implementing soil hydraulic PTFs in a spatial context

Name abbreviation	Full name	Type of data layer	Vertical coverage	Reference
SGDBE	Soil Geographical Database for Eurasia	Continuous	Topsoil and subsoil	Lambert *et al.* (2003)
HWSD	Harmonized World Soil Database	Continuous	Topsoil and subsoil	FAO/IIASA/ISRIC/ISS-CAS/JRC (2012)
GSM	GlobalSoilMap.Net	Continuous (foreseen)	Topsoil and subsoil	GlobalSoilMap.net (2011)
SPADE	Soil Profile Analytical Database for Europe	Point	Topsoil and subsoil	Hiederer *et al.* (2006)
OCTOP	Map of Topsoil Organic Carbon in Europe	Continuous	Topsoil	Jones *et al.* (2005)
LUCAS	Topsoil database of the Land Use/Cover Area frame Statistical Survey	Point (derived continuous layers foreseen)	Topsoil	Tóth *et al.* (2013)

**Figure 1:**
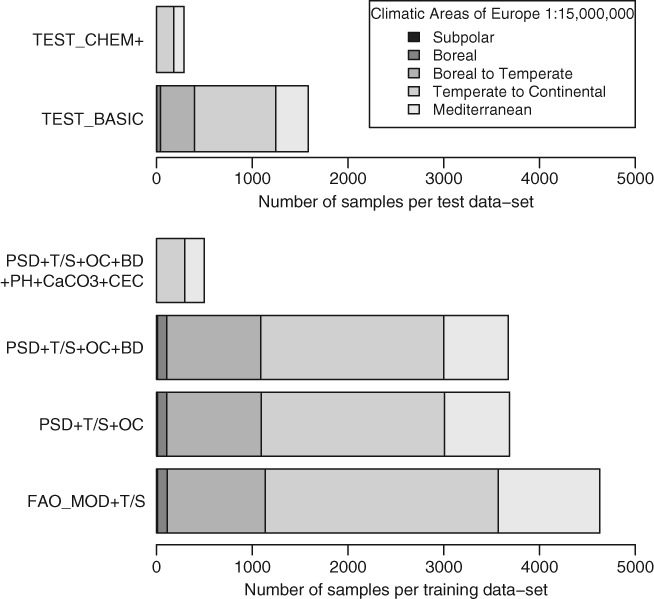
Number of samples by climatic zones (Rainer & Richter, 2005) in test and training datasets used to derive PTFs for the prediction of MRC.

The dataset, which was used for developing PTFs and assessing their reliabilities, was also derived from EU-HYDI. It was split by random sampling into training sets to derive PTFs and test sets to assess their reliability. Two types of test sets were created for each predicted hydraulic property for the comparison of derived PTFs; one for testing predictions from physical properties and organic carbon content (OC) (TEST_BASIC), and one for testing predictions using additional chemical properties (TEST_CHEM+). When additional chemical parameters were not important for a given method, the models developed were always tested against the TEST_BASIC data. Sample sizes varied according to hydraulic properties and were different for the TEST_BASIC and TEST_CHEM + test sets. A description of the data used to derive PTFs for MRC predictions is given in Table2. Figure 1 shows the number of samples by climatic zones in the training- and test datasets used to derive PTFs to predict MRC and to calculate their reliability. A summary of tested predictors and statistical approaches in training and test sets is given in Table3.

**Table 2:** Descriptive statistics of training and test sets (TEST_BASIC and TEST_CHEM+) used to derive PTFs and calculate their reliability for the prediction of the moisture retention curve (MRC)^a^

Descriptive statistic		Sand / %	Silt / %	Clay / %	Bulk density / g cm^−3^	Organic carbon / %	Calcium carbonate / %	pH in water / −
Training	N	4749	4749	4749	4830	3943	1263	1527
	Minimum	0	0	0	0.09	0	0	3.50
	Maximum	100	86.80	91.60	2.02	52.8	80	10.62
	Mean	40.82	37.25	21.92	1.39	2.9	7.92	6.98
	SD	29.00	20.63	17.09	0.29	8.0	13.01	1.19
	Median	37.06	35.20	17.80	1.44	1.0	0.40	7.10
TEST_BASIC	N	1619	1619	1619	1619	1619	288	288
	Minimum	0	0	0	0.21	0	0	4.50
	Maximum	100	84.80	88.50	1.97	33.7	65.00	10.46
	Mean	39.61	37.19	23.21	1.44	1.4	8.02	7.51
	SD	28.89	20.18	16.98	0.21	1.8	13.02	1.08
	Median	33.70	36.00	19.70	1.45	1.0	0.60	7.70
TEST_CHEM+	N	288	288	288	288	288	288	288
	Minimum	0.80	3.10	1.00	0.88	0	0	4.50
	Maximum	94.80	74.90	66.30	1.85	3.8	65.00	10.46
	Mean	37.99	37.47	24.55	1.46	1.0	8.02	7.51
	SD	25.76	17.86	13.21	0.17	0.7	13.02	1.08
	Median	33.33	36.61	23.24	1.47	0.8	0.60	7.70

a A full description of the whole EU-HYDI data-base can be found in Weynants *et al.* (2013). The TEST_BASIC dataset includes the TEST_CHEM+ dataset.

**Table 3:** Investigated input parameters for PTFs described by data source, available training and test datasets and the statistical methods tested in the development of PTFs

			Number of samples (per predicted properties^b^)						
Input parameters for PTFs^a^	Related European soil databases		*θ*_S_	*θ*_FC_	*θ*_WP_	*K*_S_	VG	MVG	Method tested^c^
FAO_MOD + T/S	SGDBE	Training	3594	2921	6074	3206	4906	860	RT
									MS
FAO_MOD + T/S + OC	SGDBE + OCTOP	Training	3204	2437	5608	2669	3943	528	RT
USDA + T/S	—	Training	3594	2921	6074	3206	4906	860	RT
									MS
PSD + T/S + OC	LUCAS/GSM/HWSD	Training	3073	2356	5530	2628	3786	407	RT
									LR
PSD + T/S + OC + pH + CaCO_3_ + CEC	LUCAS/HWSD	Training	369	657	691	401	671	135	RT
									LR
PSD + T/S + OC + BD	GSM/HWSD	Training	3065	2351	5512	2616	3773	404	RT
									LR
PSD + T/S + OC + BD + pH	GSM/HWSD	Training	1142	1933	2612	864	1713	223	RT
									LR
PSD + T/S + OC + BD + pH + CaCO_3_ + CEC	HWSD or SPADE/M or any other relevant data-set	Training	369	655	687	401	670	134	RT
									LR
(x)		TEST_BASIC^d^	1311	1005	2357	1121	1619	176	
(x)		TEST_CHEM + e	156	280	295	169	288	57	

a FAO_MOD, modified FAO texture class; T/S, topsoil and subsoil; OC, organic carbon content (100 g g^−1^); PSD, particle size distribution (sand, 50–2000 µm; silt, 2–50 µm; clay, < 2 µm (100 g g^−1^)); CaCO_3_, calcium carbonate content (100 g g^−1^); CEC, cation exchange capacity (cmol (+) kg^−1^); BD, bulk density (g cm^−3^)

b *θ*_S_, saturated water content; *θ*_FC_, water content at field capacity (pF 2.5); *θ*_WP_, water content at wilting point (pF 4.2); *K*_S_, saturated hydraulic conductivity (cm day^−1^); VG, parameters of the van Genuchten model; MVG, parameters of the Mualem – van Genuchten model.

c RT, regression tree; MS, mean statistics to derive class PTFs; LR, linear regression. Prediction of VG parameters was derived by mRT: multivariate regression tree as well.

d TEST_BASIC: samples having measured sand, silt and clay content, bulk density, topsoil/subsoil distinction and organic carbon content.

e TEST_CHEM+: samples with measured sand, silt and clay content, bulk density, topsoil/subsoil distinction, organic carbon content, pH, calcium carbonate content and cation exchange capacity.

To enable a comparison of the reliability of the new EU-HYDI and the predecessor HYPRES-based parameter estimation models, we eliminated those samples from the TEST_BASIC test set that were transferred from the HYPRES to the EU-HYDI database. This was necessary because HYPRES's models were developed on the full dataset available at the time, and reliability can only be tested on independent data.

### Predicted soil hydraulic properties

We developed point predictions to calculate moisture retention (*θ*) at three given matric potential values (*h*), namely at saturation (*θ*_S_), field capacity (*θ*_FC_) and wilting point (*θ*_WP_), and also for saturated hydraulic conductivity (*K*_S_). In addition, we made parameter estimations to describe the MRC and the HCC to provide PTFs that are applicable in a range of European- and regional-scale models.

#### Saturated water content

Saturated water content (*θ*_S_) was predicted from data measured at 0 cm matric potential.

#### Water content at field capacity

The traditional definition of water content at field capacity (*θ*_FC_) states that it is the water content that can be held against gravity 2 or 3 days after wetting the soil profile (Veihmeyer & Hendrickson, 1931). Because of this definition, *θ*_FC_ can only be approximated to a matric potential value, for which the reference value varies (−50, −60, −100 or −330 cm), depending on traditions and application needs throughout the world.

As a commonly used modelling application, the SWAT hydrological model uses −330 cm matric potential to define *θ*_FC_. Because the SWAT model is used in the MyWater project (FP7/2007–2013), into which the new PTFs feed directly, we predicted water content at this matric potential as *θ*_FC_. Should *θ*_FC_ at a different matric potential be required, we recommend that the water content at the desired matric potential is calculated from the MRC predictions, which we also describe. File S1 provides additional details on data preparation for the *θ*_FC_ predictions.

#### Water content at wilting point

We refer to the wilting point (*θ*_WP_) as the soil moisture content at −15 848 cm matric potential (pF 4.2). The measurement closest to this matric potential was chosen from the range between −15 000 and −16 000 cm, which was adjusted to equal pF4.2: this range of matric potentials is available for many samples.

#### Saturated hydraulic conductivity

We used hydraulic conductivities measured at 0 cm matric potential to predict the saturated hydraulic conductivity (*K*_S_), and used the common base logarithm of *K*_S_ (log_10_(*K*_S_)) as the dependent variable (Vereecken *et al.*, 1990; Lilly *et al.*, 2008; Weynants *et al.*, 2009).

#### Parameters of the Mualem-van Genuchten model

For the description of the full range of the moisture retention and hydraulic conductivity curve (MRC and HCC), the classic Mualem-van Genuchten model was used (MVG; Mualem, 1976; van Genuchten, 1980). For the estimation of the MRC and the HCC we predicted the *θ*_r_, *θ*_s_, *α*, *n*, *K*_0_, *L* parameters of the MVG model. Details of the calculation and basis for this model are included in File S1. The filtered data were used to fit the Mualem-van Genuchten model sequentially by using the R package *optimx* as an interface for algorithm *nlminb* (unconstrained and box-constrained optimization using PORT routines; Gay, 1990).

### Methods to build pedotransfer functions

For the targeted soil hydraulic properties, a series of pedotransfer functions (PTFs) were developed in a hierarchical approach, considering different sets of descriptive variables. Possible inputs for hydraulic predictions can be of three types: quantitative (continuous), qualitative (categorical: nominal or ordinal) or mixed (both quantitative and qualitative).

Because of the nature of the input variables, three types of prediction methods (statistical approaches) were applied to derive quantitative hydraulic properties: (i) mean statistics (MS) with qualitative independent variables, (ii) linear regression (LR) with quantitative independent variables or (iii) univariate or multivariate regression trees (RT, mRT) with quantitative, qualitative or mixed independent variables. Table3 shows the statistical approaches tested for each set of available training data. All statistical analyses were performed in R statistics, version 3.0.1 (R Core Team, 2013).

#### Mean statistics for class PTFs

When qualitative input parameters with a reasonable number of categories (classes) were available, class PTFs, referred to by Wösten *et al.* (1999) in their statistical approach as MS, were also used to predict soil hydraulic properties. For point estimations, we calculated the geometric mean value of *θ*_S_, *θ*_FC_ and *θ*_WP_ and median of log_10_*K*_S_ by soil texture classes with a topsoil/subsoil distinction (T/S) within each class.

The MSs for parameter estimations were derived by directly fitting VG and MVG to all measured *θ-h* and *K-h* data available for each combination of texture class and T/S. The objective function was the sum of all squared residuals. Class PTFs (MSs) were developed for both modified FAO (FAO_MOD) (CEC, 1985) and USDA (Soil Survey Staff, 1975) texture classes as well as organic soils, after Wösten *et al.* (1999).

#### Univariate and multivariate regression trees

A regression tree is a type of decision tree that is implemented in statistical programs as part of the Classification and Regression Trees (CART) module. In decision trees the aim is to partition the data into groups that are as homogenous as possible, in terms of the dependent variable(s). The CART module can use both continuous and categorical (ordinal or nominal) dependent and independent variables. We refer to regression trees (RTs) as those decision tree models where dependent variables are continuous-type hydraulic properties. For point predictions we built univariate RTs that provide an estimate for a single output variable. For parameter estimations, except for univariate RTs for each parameter, we also derived predictions with multivariate regression trees (mRT), which allow a joint estimation of MVG parameters that are known to be correlated. A detailed description of the application of the mRT approach is provided in File S1.

#### Linear regression

When we used continuous predictors only, along with the T/S, we fitted multiple linear regression models to the data. T/S was included in the model as a dummy independent variable with two values (topsoil = 1, subsoil = 0). Linear regression has the advantage of being easy to implement, because a unique equation results for each predicted variable, and their prediction performance was similar to regression trees for point predictions (Tóth *et al.*, 2012). Different types of linear regressions (linear regression using primary data (LR), linear regression using primary and transformed data and their interactions (LRt), and linear regression using primary and/or transformed input parameters, whichever was closest to normal distribution (LRt2)), were tested; a full description of the approach to fitting linear regression models is provided in File S1.

### Model performance measures

Performance of the developed models was characterized by their reliability, as indicated by the difference between measured and predicted values. The root mean square error (RMSE) was used for point and parameter estimations (cm^3^ cm^−3^ for water retention, log_10_ (cm day^−1^) for hydraulic conductivity). In addition, the mean error (ME) was also used for parameter estimations. To define the most reliable PTFs, simple pair-wise comparisons were performed on the MSE values of the tested PTFs. Student's *t* approach at the 5% significance level was applied using the R package *agricolae*. The reliability of the methods was computed on both the TEST_BASIC and TEST_CHEM + test sets, which are described above. File S1 provides details of their calculation.

### Principles of model selection

All combinations of available input variables and statistical prediction methods were tested during PTF development. We include here only the models that were the most reliable for the targeted soil hydraulic property, as determined by the series of tested input parameters in a hierarchical order of input requirements. The required input parameters refer to the soil properties needed to improve the estimation reliability.

In order to recommend a method for a given soil hydraulic property, a priority-based selection procedure was used. The most important criterion was the model reliability, which was chosen for each dependent variable and for combinations of input variables in a stepwise hierarchical approach. Prediction errors of PTFs were compared statistically to select the most reliable method. If no significant difference was observed between models we applied a second criterion by preferring models that use fewer input variables. We also applied a third criterion (for practical purposes) and if the number of input variables used in two models was the same, we chose that which was easier to implement, from a comparison of the computational procedure required for application. For example, a model with fewer terminal nodes was selected for PTFs based on RT. However, we gave preference to an LR-based model over an RT model because it was simpler to implement. In the case of MSs and RTs with similar reliability, preference was given to the model that included more samples in its groups/terminal nodes. If reliabilities of point estimation with RT and parameter estimation with MS of water retention were similar, we used point estimation with RT, because of its one-step straight-forward implementation.

We provide RT-based models for use with either the FAO_MOD or the USDA texture classes with T/S regardless of the above criteria because some data-sets of potential application may have one classification available but no detailed data to convert it to the other.

## Results and discussion

We performed the series of statistical analyses noted earlier and summarized the most reliable models in Table4, including their input parameters and model performance indicators. Table4 lists all tested input parameters and those ‘required’ inputs that were eventually found to be significant in the most reliable models. In many cases, using all available soil parameters did not improve the performance of the model over that with fewer input variables.

**Table 4:** Performance and input need of the most reliable methods to predict soil hydraulic properties by tested soil data combinations

Soil hydraulic property	Tested input data combination	Most reliable prediction methods (PTFs)		Reliability of PTFs			
		Required input parameters	Best performing statistical approach	TEST_CHEM+ RMSE	ME	TEST_BASIC RMSE	ME
(a) Saturated water content / *θ*_S_				/ cm^3^ cm^−3^			
				(N = 156)		(N = 1311)	
	FAO_MOD tex, T/S	FAO_MOD tex, T/S	RT	0.064	0.013	0.075	0.002
	FAO_MOD tex, T/S, OC	FAO_MOD tex, T/S, OC	RT	0.063	0.015	0.063	0.003
	USDA tex, T/S	USDA tex, T/S	RT	0.064	0.011	0.074	0.001
	Sa, Si, Cl, T/S, OC, pH, CaCO_3_, CEC	Sa, Si, Cl, T/S, OC,	RT	0.059	0.015	0.063	0.004
	Sa, Si, Cl, T/S, OC, BD	Si, Cl, T/S, OC, BD	LRt	0.028	0.015	0.034	0.002
	Sa, Si, Cl, T/S, OC, BD, pH, CaCO_3_, CEC	Si, Cl, T/S, BD, pH	LRt	0.020	−0.001	NA^a^	NA^a^
(b) Water content at field capacity / *θ*_FC_				/ cm^3^ cm^−3^			
				(N = 280)		(N = 1005)	
	FAO_MOD tex, T/S, OC	FAO_MOD tex, T/S	RT	0.069	0.008	0.063	0.005
	USDA tex, T/S	USDA tex, T/S	RT	0.064	0.002	0.058	0.002
	Sa, Si, Cl, T/S, OC, BD, pH, CaCO_3_, CEC	Si, Cl, OC,	LRt	0.058	0.003	0.055	0.003
(c) Water content at wilting point / *θ*_WP_				/ cm^3^ cm^−3^			
				(N = 295)		(N = 2357)	
	FAO_MOD tex, T/S, OC	FAO_MOD tex, T/S	RT	0.054	0.009	0.059	0.004
	USDA tex, T/S	USDA tex, T/S	RT	0.047	0.004	0.054	0.004
	Sa, Si, Cl, T/S, OC, BD, pH, CaCO_3_, CEC	Si, Cl, OC	LRt	0.043	−0.001	0.048	0.001
(d) Common logarithm of saturated hydraulic conductivity value / log_10_*K*_S_				/ log_10_(cm day^−1^)			
				(N = 169)		(N = 1121)	
	FAO_MOD tex, T/S	FAO_MOD tex, T/S	RT	1.08	0.27	1.36	0.13
	FAO_MOD tex, T/S, OC	FAO_MOD tex, T/S, OC	RT	1.11	0.02	1.05	0
	USDA tex, T/S	USDA tex, T/S	RT	1.19	0.38	1.39	−0.10
	Sa, Si, Cl, T/S, OC, BD, pH	Sa, Si, Cl, T/S, OC	RT	1.09	0.03	1.06	−0.01
	Sa, Si, Cl, T/S, OC, BD, pH, CaCO_3_, CEC	Si, Cl, T/S, pH, CEC	LR	0.90	−0.10	NA^a^	NA^a^
(e) Moisture retention curve − MRC from VG model				/ cm^3^ cm^−3^			
				(N = 288)		(N = 1619)	
	FAO_MOD tex, T/S, OC	FAO_MOD tex, T/S	MS	0.063	0.001	0.071	−0.002
	USDA tex, T/S or Sa, Si, Cl, T/S, OC	USDA tex, T/S	MS	0.058	−0.003	0.067	−0.003
	Sa, Si, Cl, T/S, OC, pH, CaCO_3_, CEC	Sa, Si, Cl, OC, pH, CEC	RT and LRt^b^	0.054	−0.007	NA^a^	NA^a^
	Sa, Si, Cl, T/S, OC, BD	Sa, Si, Cl, T/S, OC, BD	RT and LR^b^	0.054	0	0.064	—
	Sa, Si, Cl, T/S, OC, BD, pH, CaCO_3_, CEC	Sa, Si, Cl, T/S, OC, BD, pH	RT and LRt2^b^	0.046	0.017	NA^a^	NA^a^
(f) Hydraulic conductivity curve − HCC from MVG model / log_10_*K*				/ log_10_(cm day^−1^)			
				(N = 57)		(N = 176)	
	FAO_MOD tex, T/S, OC	FAO_MOD tex, T/S	MS	0.69	0.12	0.74	0.06
	USDA tex, T/S or Sa, Si, Cl, T/S, OC, BD, pH, CaCO_3_, CEC	USDA tex, T/S	MS	0.66	0.07	0.77	0.05

a On TEST_BASIC predictions requiring additional chemical properties could not be tested.

b *θ*_r_ is derived with RT, *θ*_s_, log_10_(*α*) and log_10_(*n* − 1) are predicted with LR, LRt or LRt2 accordingly.

### Findings of the point estimation study

The RMSE values of the most reliable point estimation methods varied between 0.020 and 0.075 cm^3^ cm^−3^ for *θ*_S_, 0.055 and 0.069 cm^3^ cm^−3^ for *θ*_FC_, 0.043 and 0.059 cm^3^ cm^−3^ for *θ*_WP_ and 0.90 and 1.36 log_10_(cm day^−1^) for *K*_S_, depending on the input parameters and PTF development method used. The ME values for *θ*_S_, *θ*_FC_ and *θ*_WP_ were between −0.001 and 0.015 cm^3^ cm^−3^, thus point estimations slightly under-estimated water retention values in most cases (Table4a–c). The PTFs developed for *K*_S_ usually over-estimated conductivity on the TEST_BASIC set and under-estimated it on the TEST_CHEM + set (Table4d).

We note that in the case of point predictions based on the same qualitative input parameters, the reliability of models derived by the mean statistic of developer determined groups (MS) and regression trees (RT) was not significantly different. However, the latter always contained fewer terminal nodes than there were MS groups; thus RT was simpler. Therefore we gave preference to regression trees over MSs.

The prediction of *θ*_S_ had similar reliability regardless of whether the FAO_MOD or USDA texture classes were considered in the models. Although OC improved the prediction performance if added to texture class and T/S information, the availability of particle size distribution (PSD) data (sand, 50–2000 µm; silt, 2–50 µm; clay, 0–2 µm content) provided more reliable models. Prediction of *θ*_S_ when BD was not available was the most reliable method with an RT model that has PSD, T/S and OC as inputs. When BD was available, LRt models performed better than other model types. In the absence of information on pH, the main input of the best LRt model included PSD, T/S, OC and BD, but if pH data were available, pH replaced OC in the best fitting models. As can be seen from the RMSE values of models in Table4(a), BD seems to be the most important parameter for predicting *θ*_S_, after texture or PSD information. The inclusion of T/S in all the *θ*_S_ models, as well as the significant benefit of using OC (or pH in its absence), reflects the importance of soil structure in predicting *θ*_S_: all of these properties are related to soil structure and/or aggregate stability. The pH value, together with bulk density (BD), appears to carry more information about soil structure and aggregate stability than OC content and BD (Rasiah & Kay, 1994).

In the prediction of *θ*_FC_, the most important soil information was PSD or texture class, depending on the type of information available for prediction (Tables4b, 5). Including BD did not improve the reliability of *θ*_FC_ predictions further, possibly because of our decision to use the matric potential at −330 cm to represent *θ*_FC_. At that matric potential inter-aggregate pores at sizes that strongly correlate with BD have already drained their water content. Cation exchange capacity (CEC) and pH only slightly, but not significantly, decreased the prediction errors. The *θ*_FC_ prediction reliability based on the USDA texture classes and T/S was not significantly worse than that with particle size distribution, T/S and OC content, which is in agreement with the findings of Rawls *et al.* (2003). If PSD and OC are available, we recommend using these in an LR model rather than using the RT model with USDA texture classes and T/S. The prediction reliability of *θ*_FC_ did not increase further by including parameters additional to PSD and OC.

**Table 5:** List of recommended PTFs by predicted soil hydraulic property

	Number and name of the recommended PTF					
Tested input combinations	*θ*_S_ / cm^3^ cm^−3^	*θ*_FC_ / cm^3^ cm^−3^	*θ*_WP_ / cm^3^ cm^−3^	*K*_S_ / log_10_(cm day^−1^)	MRC / cm^3^ cm^−3^	HCC / cm day^−1^
FAO_MOD + T/S	(1) FAO_MOD + T/S_**RT**_θ_S_	(7) FAO_MOD + T/S_RT_θ_FC_	(10) FAO_MOD + T/S_RT_θ_WP_	(13) FAO_MOD + T/S_**RT**_K_S_log10_	(18) FAO_MOD + T/S_**MS**_MRC	(18) FAO_MOD + T/S_**MS**_HCC
FAO_MOD + T/S + OC	(2) FAO_MOD + T/S + OC_**RT**_θ_S_			(14) FAO_MOD + T/S + OC_**RT**_K_S_log10_		
USDA + T/S	(3) USDA + T/S_**RT**_θ_S_	(8) USDA + T/S_RT_θ_FC_	(11) USDA + T/S_RT_θ_WP_	(15) USDA + T/S_**RT**_K_S_log10_	(19) USDA + T/S_**MS**_MRC	(19) USDA + T/S_**MS**_HCC
PSD + T/S + OC	(4) PSD + T/S + OC_**RT**_θ_S_	(9) PSD + OC_**LRt**_θ_FC_	(12) PSD + OC_**LRt**_θ_WP_	(16) PSD + T/S + OC_**RT**_K_S_log10_		
PSD + T/S + OC + pH + CaCO_3_ + CEC				(17) PSD + T/S + pH + CEC_**LR**_K_S_log10_	(20) PSD + OC + pH + CEC_**LRt_MRC**^a^	
PSD + T/S + OC + BD	(5) PSD + T/S + OC + BD_**LRt**_θ_S_			(16) PSD + T/S + OC_**RT**_K_S_log10_	(21) PSD + T/S + OC + BD_**LR**_MRC^a^	
PSD + T/S + OC + BD + pH	(6) PSD + T/S + BD + pH_**LRt**_θ_S_				(22) PSD + T/S + OC + BD + pH_**LRt2**_MRC^a^	
PSD + T/S + OC + BD + pH + CaCO_3_ + CEC				(17) PSD + T/S +pH + CEC_**LR**_K_S_log10_		

*θ_r_* is derived from the RT model using sand content.

a The first parts of the names of the PTFs are indicative of the input parameters required for best-case predictions, from the tested set of input combinations of column 1. Abbreviations with bold characters refer to the recommended statistical approach; abbreviations at the end of the name indicate the targeted soil hydraulic property. (Full pedotransfer functions are available in Table S1 in File S1.)

As well as estimating *θ*_FC_, the available soil information, PSD and OC, was adequate to predict *θ*_WP_ (Tables4c, 5). Inclusion of T/S, BD, pH, CaCO_3_ or CEC did not improve the reliability of the models significantly. The value *θ*_WP_ is mainly determined by particle size distribution because at around −15 000 cm of matric potential the pores are empty and only water adsorbed on the surface of soil particles can be found in the soil matrix (Rajkai *et al.*, 1981). Clay content was the most important soil property in the models, as the literature indicates (Rajkai *et al.*, 1981; Wösten *et al.*, 2001; Bruand, 2004). As Rawls *et al.* (2003) found, the more detailed information that we had about clay content (starting from FAO_MOD texture classes, then using USDA texture classes and finally percentage clay content), the better the prediction reliability became. The OC content is important because of its adsorption properties (Rawls *et al.*, 2003) (Table4c)). We also found a good correlation between the common base logarithm of CEC and *θ*_WP_ (CF = 0.607), as found by Bruand (2004). Nevertheless, the inclusion of CEC or its derivate did not improve the prediction of *θ*_WP_ significantly. The weaker impact of CEC on *θ*_WP_ prediction could be caused by CEC's relationship to the main predictors of *θ*_WP_, namely clay and OC content.

Prediction reliability of log_10_(*K_S_*) with the suggested methods was between 0.90 and 1.39 log_10_(cm day^−1^) in RMSE, which corresponds with the reliability indices determined by Lilly *et al.* (2008). In addition to soil texture or PSD, inclusion of OC content improves the prediction of log_10_(*K_S_*) significantly. Including PSD instead of FAO_MOD texture classes did not improve the prediction reliability but the number of final groups in the model decreased. Adding BD data as well as PSD, T/S and OC in the model slightly (but not significantly) decreased the prediction errors of K_S._ Using only the simple soil properties as inputs (Table4d), an RT model using PSD, T/S and OC was the most reliable method, having an RMSE of 1.05 log_10_(cm day^−1^). If CEC and pH were also considered as inputs, the LR model (including PSD, T/S, pH, CEC) had the significantly smallest prediction errors, tested on the TEST_CHEM + set (0.90 log_10_(cm day^−1^); Table4d).

### Findings of the parameter estimation study

#### Prediction of van Genuchten (VG) parameters to describe MRC

We found the MSs to be more reliable than RT when only qualitative input properties were used. The MS, using only qualitative information, was also more reliable than LR, RT and mRT with PSD, T/S and OC, that is, a mix of qualitative and quantitative inputs. These results suggest that it is important to predict VG parameters simultaneously and linked to each other. Adding CEC and either BD or pH increased the reliability of MRC predictions (Table4e). The smallest RMSE (0.046 cm^3^ cm^−3^) occurred when MRC was predicted with LR, including transformed forms of the input parameters using PSD, T/S, OC, BD and pH as inputs, without their interactions (PSD + T/S + OC + BD + pH_LRt2). Although mRT also predicts VG parameters linked to each other, it usually had the poorest reliability among the statistical methods tested when input parameters of the models were continuous and the number of samples was small in the training set.

Predicting log_10_(*θ_r_* + 1) with linear regression resulted in negative *θ_r_* values; therefore we also tested the prediction performance while forcing *θ*_r_ to be equal to 0 in the case of negative values, and as another option used *θ_r_* predicted from MSs and RTs. Based on the reliability of the MRC predictions, it appears that the best solution is using *θ_r_* derived from an RT with only two terminal nodes determined by sand content. We recommend using those two values along with LR to predict the other parameters of the MRC.

The overall ME of PTFs was between −0.007 and 0.017 cm^3^ cm^−3^ on the TEST_CHEM + set, and close to zero when calculated for the TEST_BASIC set. The prediction of water retention points by first predicting VG parameters has generally over-estimated the retained amount of water between −5 and −50 cm matric potentials and under-estimated it between −200 and −16 000 cm matric potentials (Figure S1a, File S1). When BD was included in the MRC model, the prediction's mean squared error calculated for given matric potential ranges was improved markedly between 0 and −100 cm.

#### Prediction of Mualem-van Genuchten (MVG) parameters to describe HCC

The parameters of the HCC model were developed with MS, RT and LR methods. Those with mRT are not presented because the reliability of the mRT-based models to predict MRC was typically inferior to that with the RT.

The RMSE values of the suggested PTFs calculated for log_10_(*K*) in the test datasets varied between 0.66 and 0.77 log_10_(cm day^−1^). To estimate log_10_(*K*) values, an MS that used FAO_MOD texture classes and T/S was the most reliable when tested on the TEST_BASIC dataset, but the MS using USDA texture classes and T/S had the best reliability when tested on the TEST_CHEM + dataset. For the MRC prediction, the MS based on USDA texture classes and T/S was significantly more reliable than the MS using FAO_MOD texture classes and T/S. This might be because of the more detailed texture classification of the USDA system compared with that of the FAO_MOD system. Thus the MS developed for USDA texture classes predicts the MRC and HCC better if USDA texture classes or PSD and T/S are available.

Introducing quantitative information and chemical properties into the MVG prediction did not significantly improve the prediction of the HCC. All derived prediction methods under-estimate hydraulic conductivity (Figure S1b, File S1) close to saturation and at matric potential values between −500 and −16 000 cm. The MEs of the suggested HCC predictions are shown in Figure S1(b) File S1. Under-estimation of hydraulic conductivity between 0 and −10 cm matric potential is because of the MVG parameterization of the HCC by fitting *K*_0_, as described by Schaap & Leij (2000).

There were no samples available for the silt and silty clay topsoil classes, and silt and sandy clay subsoil classes in the training dataset. To be able to apply the USDA + T/S_MS_MVG estimations also for these soil textures, we recommend using the MVG parameters of the following other classes. In the case of silty clays and sandy clays we did not distinguish MVG parameters for topsoils and subsoils. For silts there were neither topsoil nor subsoil samples available, and we recommend the use of parameters of another texture class. We considered two options for selecting the texture class that had the most similar hydraulic properties: silty clay loams or silt loams. Rajkai *et al.* (1981) found that among different particle size fractions, the fine sand fraction (50–250 µm) had the largest influence on soil water retention between 0 and −200 cm matric potentials. Furthermore, the inflection point of MRC is around −200 cm matric potential in most cases (Rajkai & Kabos, 1999). As these influence the HCC indirectly, we assumed that similarity in sand content is the most crucial factor in comparing texture classes for the HCC prediction. Therefore, we recommend that MVG parameters of the silty clay loam topsoil and subsoil classes are used for the silt classes, because those classes have the same range of sand content (0–20%).

### Eu-hydi vs. Hypres parameter estimations

To predict the MRC, the MS (class PTFs) developed from EU-HYDI performed significantly better than the similar-type HYPRES class PTFs. The former had an overall RMSE value of 0.067 cm^3^ cm^−3^, and the latter a value of 0.072 cm^3^ cm^−3^ when tested on the TEST_BASIC set, which excluded the samples originating from HYPRES. Although continuous PTFs developed from the EU-HYDI and HYPRES with the same input parameters were not significantly different (RMSE = 0.055 and 0.056 cm^3^ cm^−3^, respectively), the PTFs presented here have the advantage of using null values as input variables, which is not the case for HYPRES continuous PTFs. Furthermore, continuous PTFs based on EU-HYDI can be also developed for cases where additional chemical property data (pH and CEC) are available. The MRC models with additional chemical information performed significantly better (RMSE = 0.046 cm^3^ cm^−3^) than those of HYPRES, which were based on texture and T/S only. This allows potentially more accurate predictions, with the use of less commonly available inputs, when those are present.

Unsaturated hydraulic conductivity predictions based on EU-HYDI MSs (class PTFs) were significantly better than the HYPRES class and continuous PTFs, with values for RMSE of 0.75, 0.96 and 0.89 log_10_(cm day^−1^), respectively.

### Comparison of point and parameter estimations

To compare the prediction power of point and parameter estimations we used the directly predicted *θ*_S_, *θ*_FC_ and *θ*_WP_ with their indirect prediction with the VG parameter estimation model. We compared the reliability of the best point estimation methods (Table4a–c) with the best parameter estimations (Table4e).

With all input combinations, we recommend that *θ*_S_, *θ*_FC_ and *θ*_WP_ are predicted with point estimation. No significant difference was found between point and parameter estimation by using FAO_MOD + T/S or USDA + T/S. However, the direct point prediction is simple to implement, while calculating water retention values from the VG model requires first the application of MRC PTF and then calculating water retention with derived VG parameters for given matric potentials. If PSD data were available, point predictions were significantly more reliable in most cases than parameter estimations. There were some cases when no significant difference was found and parameter estimation was never significantly better than point estimation. This is in agreement with Børgesen & Schaap (2005), who found, in most cases, greater RMSEs for parameter estimations than for point estimations when predicting water retention at distinct matric potential values. Parameter estimations rely on fitted parameters, and therefore always have uncertainty in their goodness of fit and have greater prediction errors than point estimations (Børgesen & Schaap, 2005).

The reliability of *K*_S_ point estimations (Table4d) and the estimation of *K*_0_ from MVG (Table4f) were not compared. Although *K*_0_ is a matching point at saturation in the description of unsaturated hydraulic conductivity, it is not necessarily equal to *K*_S_ but it is often smaller by about one order of magnitude (Schaap & Leij, 2000). Therefore the MVG model with fitted *K*_0_ is not suitable for model flows at full saturation, where it can be affected by macropores (van Genuchten & Nielsen, 1985; Schaap *et al.*, 2001). For the prediction of *K*_S_ we give preference to point predictions rather than using the MVG parameter *K*_0_.

### Importance of soil properties in estimating soil hydraulic characteristics

Generally, soil texture or PSD and T/S, OC and BD were the most important input parameters to predict soil hydraulic properties. In fact, PSD or texture class information in combination with the OC content or T/S information provides an adequate basis for the prediction of soil water status in most cases. Additional soil properties were included only in a few cases, as in the studies of Rawls *et al.* (1991) and Wösten *et al.* (2001). Thus, the texture type, or PSD if available, was by far the most important factor in describing soil water retention (Tables4a–c,e, 5). Nevertheless, other soil properties can also play significant roles, such as BD for the prediction of *θ*_S_ or MRC. The OC content is important information needed to describe soil water properties because it influences a number of other physical and physico-chemical soil properties. Mineral soils with greater OC content tend to have better soil structure, and thus increased water-holding properties, and organic matter itself also has good water-absorption properties. Nevertheless, the indirect effect of OC content on water retention through soil structure requires further investigations in the future. Information on the position of the soil in the profile (T/S) improves the predictions of most of the hydraulic properties as well. The importance of PSD or texture class information, OC content and information on whether a sample is from the topsoil or subsoil is demonstrated by their larger weighting in the prediction algorithms of LR models and greater variable importance in RT models (Table4).

The importance of BD varied according to the predicted soil hydraulic properties. It significantly improved prediction of *θ*_S_ and MRC, but was not important for the estimation of *θ*_FC_, *θ*_WP_, *K*_S_ and HCC, as shown by Børgesen & Schaap (2005) when related to water-retention predictions and Lilly *et al.* (2008) for PTFs that predict *K*_S_. The BD is, however, also influenced by other input variables that may also be available.

Additionally, soil chemical properties that we studied (pH, CaCO_3_ and CEC) were also important for prediction of some of the soil hydraulic properties, such as *θ*_S_, MRC and *K*_S_. However, they did not improve *θ*_FC_ and *θ*_WP_ point predictions significantly or estimation of the HCC parameter. Information on pH and CEC improved the prediction reliability of *K*_S_ and the MRC: pH might influence soil structure (Hodnett & Tomasella, 2002; Tóth *et al.*, 2012), which is closely correlated to water retention close to saturation and saturated hydraulic conductivity, and CEC is related to the clay mineralogy and forms of organic matter (Bruand, 2004; Botula Manyala Ilanga *et al.*, 2013).

As well as the chemical and physical soil properties as inputs, the measurement technique used to determine hydraulic conductivity may also influence the quality of *K*_S_ and HCC predictions (Vereecken, 2002). In EU-HYDI, both the sample sizes and measurement methods were very diverse (Weynants *et al.*, 2013), especially for hydraulic conductivity. Thus the correlation between basic soil properties and hydraulic conductivity may not be clear from the data subset used to derive PTFs. This may be a reason why the very simple MSs were the most reliable method for HCC prediction even though they were based on textural information and T/S only. The reliability of estimation may improve if the data (if the quantity allows) are pre-sorted by measurement methods and measurement-method-specific PTFs are developed.

### Model recommendation

Table5 lists recommended methods, and also considers different levels of input availability. From our discussion of model selection principles, point estimation is preferred for prediction of *θ*_S_, *θ*_FC_ or *θ*_WP_. On the other hand, if water content at matric potential other than 0, −330 and −15 000 cm is needed we recommend that parameter-based PTFs are used.

However, parameter estimation with MSs is recommended when only texture is available. If PSD is available, different models are recommended depending on the soil hydraulic property required and the input parameters available for the prediction. Prediction of the common base logarithm of *K_S_* is better when using point estimations than its calculation from the HCC predictions.

If the reliability of both the MRC and the HCC is important, the USDA + T/S_MS_HCC model based on the prior development of the USDA + T/S_MS_MRC model is the most reliable method. If only the FAO_MOD texture-class information is available, the recommendation changes to the FAO_MOD + T/S_MS_HCC model based on prior development of the FAO_MOD + T/S_MS_MRC model. If the reliability of the HCC is more important than that of MRC, we recommend MS (class PTF) based on FAO_MOD texture classes and T/S, or the USDA texture classes and T/S if that is the only textural classification that is available. All recommended PTFs are included in Table S1 in File S1. An open source R software package was developed to assist the implementation of PTFs presented in this article and can be accessed at the European Soil Data Centre (http://eusoils.jrc.ec.europa.eu/).

## Conclusions

We developed PTFs for continental-scale applications in Europe for a series of potential needs, and we adapted a hierarchical approach that facilitates their use with the most commonly used spatial soil datasets of the continent. The results of our model development provide significant improvement in the reliability of European-scale PTFs when the most traditional input variables are used. In addition, the importance of chemical variables (CaCO_3_, CEC and pH), which were not considered in earlier predictions on this scale in Europe, was demonstrated when predicting *θ*_S_, *K*_S_ and MRC parameters. Differences in the adequacy of various statistical methods for developing input- and output-specific soil hydraulic models were demonstrated for a large, continental dataset, highlighting the need for purpose-specific statistical procedures in soil hydraulic research. To serve user needs, the best fitting, purpose-specific models are recommended and shown by hierarchically structured combinations of input parameters. Derived soil hydraulic PTFs enable the preparation of a series of reliable soil hydraulic maps for Europe using different sets of soil map information.

For further improvement of the models it is desirable to provide a better representation of soils of Europe by including more silty soils and sandy clay soils in the EU-HYDI in the future. It is noted, however, that scarcity of silty and sandy clay samples in the database is primarily driven by their natural scarcity in Europe, because of its climatic and geological conditions. While the PTFs presented quantify the dependency of water retention and conductivity characteristics on basic soil properties in Europe with better accuracy and reliability than previous models, special-case PTFs, such as those based on specific regions, soil types, measurement methodology or land use and soil management, will further improve predictions. Therefore, it is desirable that the collection and analysis of soil hydraulic datasets that are relevant for these special aspects are improved.

It is noted that prediction accuracy of a soil hydraulic property in any spatial application does not only depend on the accuracy of the used PTF but also on the quality of underlying data, including the spatial accuracy of the map on which it is implemented.

## Supporting Information

The following supporting information is available in the online version of this article:

File S1 A new generation of hydraulic pedotransfer functions for Europe.

## References

[b1] Adhikari A, Kheir RB, Greve MB, Bøcher PK, Malone BP, Minasny B (2013). High-resolution 3-D mapping of soil texture in Denmark. Soil Science Society of America Journal.

[b2] Arrouays D, Grundy MG, Hartemink AE, Hempel JW, Heuvelink GBM, Hong YS (2014). GlobalSoilMap: toward a fine-resolution global grid of soil properties. Advances in Agronomy.

[b3] Baker L, Ellison D (2008). Optimisation of pedotransfer functions using an artificial neural network ensemble method. Geoderma.

[b4] Børgesen CD, Schaap MG (2005). Point and parameter pedotransfer functions for water retention predictions for Danish soils. Geoderma.

[b5] Botula Manyala Ilanga YD, Nemes A, Mafuka P, Van Ranst E, Cornelis W (2013). Prediction of water retention of soils from the humid tropics by the nonparametric k-nearest neighbor approach. Vadose Zone Journal.

[b6] Bruand A, Rawls WJ, Pachepsky Y (2004). Utilizing mineralogical and chemical information in PTFs. Developments in Soil Science.

[b39] Commission of the European Communities, CEC (1985).

[b7] FAO/IIASA/ISRIC/ISS-CAS/JRC (2012). Harmonized World Soil Database (Version 1.2).

[b8] Gay DM (1990).

[b11] (2011). http://GlobalSoilMap.net.

[b12] Hiederer R, Jones RJA, Daroussin J (2006). Soil Profile Analytical Database for Europe (SPADE): reconstruction and validation of the measured data (SPADE/M). Geografisk Tidsskrift.

[b13] Hodnett MG, Tomasella J (2002). Marked differences between van Genuchten soil water-retention parameters for temperate and tropical soils: a new water-retention pedo-transfer functions developed for tropical soils. Geoderma.

[b14] Jones RJA, Hiederer R, Rusco E, Loveland PJ, Montanarella L (2005). Estimating organic carbon in the soils of Europe for policy support. European Journal of Soil Science.

[b15] Lambert JJ, Daroussin J, Eimberck M, Le Bas C, Jamagne M, King D (2003).

[b16] Lilly A, Nemes A, Rawls WJ, Pachepsky Y (2008). Probabilistic approach to the identification of input variables to estimate hydraulic conductivity. Soil Science Society of America Journal.

[b17] Mualem Y (1976). A new model for predicting the hydraulic conductivity of unsaturated porous media. Water Resources Research.

[b18] Nemes A, Schaap M, Wösten J (2003). Functional evaluation of pedotransfer functions derived from different scales of data collection. Soil Science Society of America Journal.

[b19] Pachepsky Y, Timlin D, Várallyay G (1996). Artificial neural networks to estimate soil water retention from easily measurable data. Soil Science Society of America Journal.

[b20] Rainer B, Richter S (2005). http://www.bgr.de/app/FISBoBGR_Produktauswahl/Produktkatalog/metadata.php?id=146&lang=en&pid=1.

[b21] Rajkai K, Kabos S (1999). Estimation of soil water retention characteristics (pF curves) from other soil properties. (A talaj víztartóképesség-függvény (pF-görbe) talajtulajdonságok alapján történő becslésének továbbfejlesztése.). Agrokémia és Talajtan.

[b22] Rajkai K, Várallyay G, Pachepsky Y, Scherbakov RA (1981). Calculation of water retention data from the texture and the bulk density of soils. (pF-görbék számítása a talaj mechanikai összetétele és térfogattömege alapján.). Agrokémia és Talajtan.

[b23] Rasiah V, Kay BD (1994). Characterizing changes in aggregate stability subsequent to introduction of forages. Soil Science Society of America Journal.

[b24] Rawls WJ, Gish TJ, Brakensiek DL (1991). Estimating soil water retention from soil physical properties and characteristics. Advances in Soil Science.

[b25] Rawls W, Pachepsky Y, Ritchie J (2003). Effect of soil organic carbon on soil water retention. Geoderma.

[b26] R Core Team (2013). R: A Language and Environment for Statistical Computing.

[b27] Schaap GM, Leij FJ (2000). Improved predictions of unsaturated hydraulic conductivity with the Mualem-van Genuchten model. Soil Science Society of America Journal.

[b28] Schaap MG, Leij FJ, van Genuchten MT (2001). Rosetta: a computer program for estimating soil hydraulic parameters with hierarchical pedotransfer functions. Journal of Hydrology.

[b40] Soil Survey Staff (1975).

[b29] Tóth B, Makó A, Guadagnini A, Tóth G (2012). Water retention of salt affected soils: quantitative estimation using soil survey information. Arid Land Research & Management.

[b30] Tóth G, Jones A, Montanarella L (2013). The LUCAS topsoil database and derived information on the regional variability of cropland topsoil properties in the European Union. Environmental Monitoring & Assessment.

[b9] van Genuchten MT (1980). A closed-form equation for predicting the hydraulic conductivity of unsaturated soils. Soil Science Society of America Journal.

[b10] van Genuchten MT, Nielsen DR (1985). On describing and predicting the hydraulic conductivity of unsaturated soils. Annales Geophysicae.

[b31] Veihmeyer FJ, Hendrickson AH (1931). The moisture equivalent as a measure of the field capacity of soils. Soil Science.

[b32] Vereecken H (2002). Comment on the paper, “Evaluation of pedo-transfer functions for unsaturated soil hydraulic conductivity using an independent data set”. Geoderma.

[b33] Vereecken H, Maes J, Feyen J, Darius P (1989). Estimating the soil moisture retention characteristic from texture, bulk density, and carbon content. Soil Science.

[b34] Vereecken H, Maes J, Feyen J (1990). Estimating unsaturated hydraulic conductivity from easily measured soil properties. Soil Science.

[b35] Weynants M, Vereecken H, Javaux M (2009). Revisiting vereecken pedotransfer functions: introducing a closed-form hydraulic model. Vadose Zone Journal.

[b36] Weynants M, Montanarella L, Tóth G, Strauss P, Feichtinger F, Cornelis W (2013). EUR – Scientific and Technical Research Series. European HYdropedological Data Inventory (EU-HYDI).

[b37] Wösten JHM, Lilly A, Nemes A, Le Bas C (1999). Development and use of a database of hydraulic properties of European soils. Geoderma.

[b38] Wösten JHM, Pachepsky Y, Rawls WJ (2001). Pedotransfer functions: bridging the gap between available basic soil data and missing soil hydraulic characteristics. Journal of Hydrology.

