# Supplementary material for: New generation of hydraulic pedotransfer functions for Europe
Source: Eur J Soil Sci. 2014 Nov 3;66(1):226–38. doi: 10.1111/ejss.12192 (PMC4386477; doi:10.1111/ejss.12192)
Supplement: File S1 — A new generation of hydraulic pedotransfer functions for Europe. [file ejss0066-0226-sd1.docx]

A new generation of hydraulic pedotransfer functions for Europe

B. Tóth^a^, M. Weynants^b^, A. Nemes^c^, A. Makó^d^, G. Bilas^e^ & G. Tóth^b^

**SUPPLEMENTARY MATERIAL**

**Supplementary information on data preparation and statistical approaches**

*Data preparation*

The information available for each sample in the EU-HYDI varies and chemical, physical and hydraulic data were in many cases determined with different methods because of different standards applicable at the data providers’ institutions. Moisture retention and hydraulic conductivity data in EU-HYDI were measured with different methods. These included field or laboratory measurements or the combination of both. Sample sizes varied among the data sources. Harmonization of particle size fraction limits and organic carbon content was carried out (Weynants *et al.*, 2013). We checked each samples to see if their water retention values decreased monotonously with decreasing matric potential. Part of the moisture retention curves was visually controlled and unreliable data was flagged. Uncharacteristic information was filtered out before our analysis. Texture classes were based on the modified FAO (FAO_MOD) (CEC, 1985) and USDA texture classes, with the addition of organic class after Wösten *et al.* (1999). For other measurements, harmonization was not performed because there are no sound correction measures. A lack of harmonization can lead to a decreased uniformity of the data-set and a reduced accuracy of pedotransfer functions derived from the measured data (Batjes, 2009).

We split the data-base by hydraulic properties to training and test sets. The goal of dividing the whole data-set into training and test sets was to achieve comparability among the tested PTFs. Results obtained by different statistical approaches should be compared on the same test-data set, even if predictions are made from training sets of different sample sizes or by using a different set of input variables in the same method.

Test sets contained randomly selected data amounting to 30% of the training set which had the fewest samples available to predict the given hydraulic property from the input parameters of interest. Sample sizes in the training data-sets also varied, but always contained at least 70% of samples eligible for the given prediction. Inclusion of samples from all USDA and FAO_MOD texture classes in the training sets were ensured, whenever possible.

The top-soil and sub-soil distinction of samples in the EU-HYDI was based on horizon names and sampling depth. Those samples of the database which were topmost A or O or H horizons or transitional horizons of A starting at 0 cm depth were considered as top-soil samples. Where there was no information about horizon designation in the data-set, layers with 0 cm top depth or samples that were entirely from the 0–30cm depth and whose top was within 0–5 cm depth were considered to represent the top-soil. The rest of the samples were considered as sub-soil samples. The 30-cm lower boundary for the top-soil was set in accordance with IPCC recommendations (IPCC, 2003) and as general practice for characterizing upper soil layers in Europe (Jones *et al.,* 2005).

Soil hydraulic data were fitted with the Mualem-van Genuchten (MVG) model (Equations (S1), (S2) and (S3)). The model parameter boundaries were established as follows: *θ*_r_ ∈ [0; 0.35], *θ*_s_ ∈ [0.2; 0.85], *α* ∈ [0.00001; 0.99999], *n* ∈ [1.001; 15], *K*_0_ ∈ [0.001; 10000] and *L* ∈ [-5; 5]. Water retention values measured at matric potential smaller than -16 000 cm were not considered while fitting the model. The van Genuchten water retention model can be written as:

$\theta\left( h \right)=\theta_{r}+\frac{\theta_{s}-\theta_{r}}{\left[ {1+\left( \alpha h \right)}^{n} \right]^{m}}$, (S1)

where *θ*(*h*) is the water content of the soil (cm³ cm^-3^) at a given matric potential value (cm of water column); *θ_r_* is the residual water content (cm³ cm^-3^); *θ_s_* is the saturated water content (cm³ cm^-3^); and *α* (cm^-1^), *n* (-), and *m* (-) are fitting parameters

The van Genuchten model can be coupled with the model of Mualem (1976) which describes the hydraulic conductivity curve (HCC). Provided that *m*=1-1/*n* (van Genuchten, 1980), it can be written as

$K\left( S_{e} \right)=K_{0}S_{e}^{L}\left[ {1-\left( 1-S_{e}^{1/m} \right)}^{m} \right]^{2}$, (S2)

$S_{e}\left( h \right)=\frac{\theta\left( h \right)-\theta_{r}}{\theta_{s}-\theta_{r}}$, (S3)

where *K* is the soil hydraulic conductivity (cm day^-1^); *K_0_* is the hydraulic conductivity acting as a matching point at saturation (cm day^-1^); *S_e_* is the effective saturation (-) and *L* is a shape parameter related to pore tortuosity (-).

We estimated the parameters of the classic MVG model in our study despite its documented weaknesses. Our decision was determined because this approach is employed by the currently available simulation models. The classic MVG model may under-estimate the hydraulic conductivity close to saturation (Schaap & Leij, 2000) and may lead to unstable numerical solutions of the Richards equation (Richards, 1931) when parameter *n* is less than 2 (Ippisch *et al.*, 2006). The introduction of an air-entry point in the MVG model improves the description of the HCC but not that of the MRC (Schaap & van Genuchten, 2006; Weynants *et al.*, 2009). The modified model by Ippisch *et al.* (2006) needs a further parameter to those of the classic MVG and the modification suggested by Schaap & van Genuchten (2006) is data-base specific. Since not all environmental models have the modified MVG equations implemented, we developed PTFs to predict the parameters of the classic model (*θ*_r_, *θ*_s_, *α*, *n*, *K*_0_, *L*).

In EU-HYDI, the methods used to measure the soil hydraulic properties are diverse. Hence, the available number of data pairs [(*θ*, *h*) or (*K*, *h*)] for each sample can range from 1 up to several hundred when evaporation methods were used. Therefore, before fitting any model to these data, we filtered both training and test sets according to the following rules: (i) we considered only samples with at least five data pairs; (ii) for samples with more than 30 data pairs, we kept the first, that closest to saturation, and randomly selected up to two points within ranges between the following matric potential values: 0, -10, -20, -50, -100, -200, -250, -500, -1000, -2000, -5000, -10000, -15 000 and -16 000 cm. Using a similar number of data pairs for all samples reduced the bias towards samples with many observations in the calculation of the errors. For example, samples based on evaporation data had thousands of observations whereas others (such as from combination of sand/kaolin box and pressure plate measurements) had only five data pairs. The filtered data was then used to fit the MVG model. First, the van Genuchten model with *m* =1–1/*n* was fitted to the retention data. Then, for samples with conductivity data, both *K*_0_ and *L* were fitted, using the *α* and *n* values obtained from the retention data. The MRC was fitted for samples with at least five *θ*-*h* pairs. The HCC was subsequently fitted for samples for which the MRC could be fitted and at least five *K*-*h* pairs were available. The objective function to be minimized was the sum of squared residuals between observations and estimations. Given the skewed distribution of the hydraulic conductivity, the common base logarithm was used both as fitting parameter (log_10_(*K*_0_)) and in the objective function (Schaap & Leij, 2000). Although the algorithm converged in most cases, the parameters reached the boundaries in many cases. Only the samples for which the algorithm did converge were kept for the PTFs’ development (Figure 1 of the main text).

In EU-HYDI, many soil samples had water retention measurements at -330 cm matric potential, which is often considered as the laboratory equivalent for field capacity. However, numerous other samples did not have measurements available at exactly this value. To minimize this problem, only samples having measurements in between -300 to -350 cm matric potential (*h*) were selected for the predictions. For these samples, three criteria were then set: (i) either one measurement was required at exactly -330 cm, or (ii) one measurement smaller and one larger than -330 cm was required, or (iii) at least one measurement in the range between -282 and -354 cm (for which pF (log(-*h*)) can be rounded to 2.5) was required. For samples satisfying the first criterion, no approximation was needed (42% of available samples). For those satisfying the second criterion but not the first (6% of available data) a linear interpolation between the two closest measurements smaller and greater than -330 cm was made. We also tested calculating them with the van Genuchten model (Equation (S1)), after fitting the model on measured *θ*-*h* pairs, but we did not use this option because the linear approximation gave better results. For samples satisfying the third, but not the first two, criterion (52% of the samples), the measurement closest to -330 cm in the specified range was used as an approximation of field capacity. Samples that did not satisfy any of the three criteria were not considered for this part of the analysis.

We used logarithmic transformations of *θ*_r_, *α*, *n* and *K*_0_ (log_10_(*θ*_r_+1), log_10_(*α*), log_10_(*n*-1), log_10_(*K*_0_)) in the MVG parameter prediction (Vereecken *et al*., 1989; Wösten *et al*., 1999) and of *K*_S_ (log_10_(*K*_S_)) in the point estimation (Lilly *et al*., 2008) as dependent variables.

*Statistical approaches*

Although the outcomes of class PTFs and the continuous PTF developed by RT were similar, the way the grouping of the data-set was achieved was different. Traditionally the groups (classes) of a class PTF are developer-determined, and whether all separated groups improve the model performance is not tested. The grouping in a tree-based PTF is data-driven. Although in class PTFs, MVG parameters are linked to each other, in univariate tree-based PTFs those are predicted independently from each other. However, in order to predict MVG parameters linked to each other, we also examined the prediction performance of multivariate regression trees.

All predictions were tested by using RT. We chose this method because it can describe both linear and non-linear interactions, is robust to outliers, can handle both quantitative and qualitative independent variables and is easy to interpret and implement (Breiman *et al.*, 1984). It is also a well-established method in soil hydraulic research (McKenzie & Jacquier, 1997; Rawls *et al.*, 2003; Lilly *et al.*, 2008).

To predict parameters of the MVG model, the prediction methods were set up in the following ways. For cPTFs we fitted the MVG function to *K*-*h* pairs of the individual samples and grouped and averaged them by soil texture classes and top-soil and sub-soil distinction, by using VG parameters from VG class PTFs (separately for FAO_MOD and USDA). For RT and LR, we built models with dependent variables K_0_ and L that were optimized on the observed conductivity curves under the constraint of alpha and n obtained by the VG PTFs. The parameters are not independent from one another and using the values of K_0_ and L that have been optimized with a set of α and n values different than that used when using the PTFs was not sensible.

*Basic principles for fitting a multivariate regression tree (mRT) model* In mRT, as for RT, the objective was to maximize the homogeneity of the data at the terminal nodes and minimize the total impurity of terminal nodes (De’ath, 2002). The RTs and mRTs were built with the mvpart package of R. The model uses the least-squared deviation measure of impurity to choose the best split on the independent variable and best variable for the splitting. This criterion can be written as follows:

$R\left( t \right)=\frac{1}{N(t)}\sum_{i=1}^{N(t)} \left( y_{i}-\bar{y}_{t} \right)^{2}$ , (S4)

where *R*(*t*) is the residual sum of squares in node t, *N*(*t*) is the number of cases in node t, *y_i_* is the value of the dependent variable, and *ȳ_t_* is the mean of all values of the dependent variable in node *t*. In mRT, the multivariate sum of squared deviations about the mean was used to calculate the impurity. The algorithm selects the independent variable’s split that gives the largest decrease in residual sum of squares. The model accuracy is characterized by the residual sum of squares calculated for all terminal nodes of the tree (Breiman *et al.,* 1984; De’ath, 2002).

To prevent over-fitting of the predictive models and incorporating data-subset specific random noise into the model instead of modelling the underlying relationship, we built a sequence of trees based on 100 ten-fold cross-validations. To build the model we used different values of the cost-complexity criterion (*c_p_*), starting from a very large tree (because of a small value of *c_p_*) to having no split of the data (because of a large value of *c_p_*). The developed regression trees were pruned by applying the 1 standard error (SE) rule (Breiman *et al.*, 1984) on the 100 times cross-validated sequence of trees. We optimized the *c_p_* parameter to produce the smallest tree with the average residual sum of squared errors (*R*). The optimization was considered to be complete when the cross-validated test data set reached a R value within one standard deviation of its minimum value. In order to provide a single pruned tree, we rebuilt the tree on the whole training set by using the optimal model settings that resulted after tuning *c_p_*.

*Basic principles for fitting the linear regression (LR) model* For each predicted variable, we developed 100 linear regression equations by stepwise regression using R functions *lm* and *step* from package *stats*. Each equation was developed on 80% of the full training set and was randomly selected. Starting with only an intercept, at each step, the algorithm calculates the Akaike Information Criterion (AIC) for including each predictor, or excluding each of those already in the model. It selects the new model which helps decrease AIC the most. The algorithm stops when no further step can make a significant improvement, or when it reaches the maximum scope, including potentially using all the predictors. Among the 100 resulting models, we looked for the most frequently occurring number of predictors by the stepwise procedure and set it as the number of terms, *n*, in the final model. Next, we examined the frequency of each predictor being included in the first *n* selected terms. We then set the *n* most frequent predictors as input for the final model, regardless of the order in which they were included by the stepwise procedure. Finally, we fitted a linear model on 1000 re-sampled sets, with 80% of the training data in each re-sampled set. Each coefficient presented in the final model is the median of the 1000 values obtained on the re-samples.

Among the textural groups, sand and clay content had the largest absolute correlation with each other but clay was more correlated with soil hydraulic properties; therefore sand content was not included in the linear regression models. Calcium-carbonate content values were highly variable, making it a near-zero-variance predictor when the data-set was split in the cross-validation scheme, and thus we did not use it in the LR model.

As well as including raw independent variables (silt, clay, organic carbon, BD, pH and CEC) in the LR model, we tested how the reliability of the prediction changes if transformation and interactions of those soil properties such as with ten-based logarithmic, reciprocal, or squared transformations of the variables were also included in the LRt model. Multi-collinearity should be prevented in linear models. Therefore, in the LRt2 model we kept only one type of independent variables for each soil input parameter (either with or without transformation) and always that having its distribution closest to normality. Normality was assessed with a visual examination of the Q-Q plot, and taking into account values of Skewness and Kurtosis. We used the selected independent variables and top-soil/sub-soil distinction in the linear model (LRt2). We kept the best of the three possible linear models (LR, LRt, LRt2) for further model comparison.

*Statistics to measure model performance* A global root mean square error (RMSE) was calculated both for point and parameter estimations as:

$RMSE=\sqrt{\frac{1}{N}\sum_{i=1}^{N} \left( y_{i}-\hat{y}_{i} \right)^{2}}=\sqrt{MSE}$. (S5)


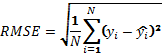


In addition, the mean error (ME) was also calculated for parameter estimations using Equation (6 ) to analyse the prediction error by matric potential value ranges between 0, -5, -10, -20, -50, -100, -200, -250, -500, -1000, -2000, -5000, -15 000 and -16 000 cm as:

$ME=\frac{1}{N}\sum_{i=1}^{N} \left( y_{i}-\hat{y}_{i} \right)$, (S6)

where *y_i_* is the measured soil water content or logarithmic transformation of saturated or unsaturated hydraulic conductivity, *ŷ_i_* the predicted soil water content or logarithmic transformation of saturated or unsaturated hydraulic conductivity, *N* the number of *y_i_* and *ŷ_i_* data pairs and MSE the mean squared error. To test the reliability of parameter estimations (MRC and HCC), values of *θ*(*h*) and *K*(*h*) were calculated with predicted MVG parameters and then compared with the original measured *θ*(*h*) and *K*(*h*) values in the data-set for matric potential values that were available.

*Mean error of recommended MRC and HCC predictions by matric potential values.*

**Figure S1a** MRC predictions (calculated for the TEST_CHEM+ set, N = 288)

**Figure S1b** HCC prediction (calculated for the TEST_BASIC set, N = 176)

*Recommended prediction models for regional to continental scale applications in Europe*

**Table S1** Suggested prediction methods to use for a given set of available input parameters per each examined soil hydraulic property)^a^.

| **Number of model indicated in Table 5** | **Predicted soil hydraulic property** | **Type of model** | **Prediction model** |
| --- | --- | --- | --- |
| (1) | *θ*_S_  / cm^3^cm^-3^ | RT | Rule 1  IF FAO_MOD=coarse,medium,medium fine AND T/S=sub  θ_S_ =0.416  Rule 2  IF FAO_MOD=coarse,medium,medium fine AND T/S=top  θ_S_ =0.467  Rule 3  IF FAO_MOD=fine  θ_S_ =0.475  Rule 4  IF FAO_MOD=very fine  θ_S_ =0.564  Rule 5  IF FAO_MOD=organic  θ_S_ =0.847 |
| (2) | *θ*_S_  / cm^3^cm^-3^ | RT | Rule 1  IF FAO_MOD=coarse,medium,medium fine AND OC< 0.11  θ_S_ =0.365  Rule 2  IF FAO_MOD=coarse,medium,medium fine AND OC>=0.11 AND OC< 0.17  θ_S_ =0.393  Rule 3  IF OC>=0.17 AND OC< 1.74 AND FAO_MOD=coarse  θ_S_ =0.399  Rule 4  IF FAO_MOD=medium AND OC>=0.90 AND OC< 1.74 AND T/S=sub  θ_S_ =0.389  Rule 5  IF FAO_MOD=medium AND OC>=0.90 AND OC< 1.74 AND T/S=top  θ_S_ =0.435  Rule 6  IF OC>=0.17 AND OC< 0.90 AND FAO_MOD=medium  θ_S_ =0.427  Rule 7  IF OC>=0.17 AND OC< 0.32 AND FAO_MOD=medium fine  θ_S_ =0.412  Rule 8  IF FAO_MOD=medium fine AND OC>=0.32 AND OC< 1.74  θ_S_ =0.453  Rule 9  IF FAO_MOD=fine,very fine AND OC< 0.63  θ_S_ =0.434  Rule 10  IF OC>=0.63 AND OC< 1.74 AND FAO_MOD=fine  θ_S_ =0.482  Rule 11  IF OC>=0.63 AND OC< 1.74 AND FAO_MOD=very fine  θ_S_ =0.557  Rule 12  IF OC>=1.74 AND OC< 2.21 AND FAO_MOD=coarse,fine,medium  θ_S_ =0.461  Rule 13  IF OC>=3.79 AND OC<4.64 AND T/S=top AND FAO_MOD=fine  θ_S_ =0.377  Rule 14  IF OC< 3.79 AND OC>=1.74 AND T/S=top AND FAO_MOD=fine  θ_S_ =0.471  Rule 15  IF OC>= 2.21 AND OC<4.64 AND T/S=top AND FAO_MOD=coarse,medium  θ_S_ =0.488  Rule 16  IF OC>= 2.21 AND OC<4.64 AND T/S=sub AND FAO_MOD=coarse,medium  θ_S_ =0.477  Rule 17  IF OC>= 2.21 AND OC<4.64 AND T/S=sub AND FAO_MOD=fine  θ_S_ =0.570  Rule 18  IF FAO_MOD=medium fine AND OC< 3.11 AND OC>=1.74  θ_S_ =0.502  Rule 19  IF FAO_MOD=medium fine AND OC>=3.11 AND OC<4.64  θ_S_ =0.596  Rule 20  IF OC>=1.74 AND OC<4.64 AND FAO_MOD=very fine  θ_S_ =0.628  Rule 21  IF OC>=4.64 AND OC< 7.85 AND FAO_MOD=coarse,medium  θ_S_ =0.531  Rule 22  IF FAO_MOD=coarse,medium AND OC>=7.85 AND OC<10.89  θ_S_ =0.594  Rule 23  IF OC>=4.64 AND OC<10.89 AND FAO_MOD=fine,medium fine,very fine  θ_S_ =0.625  Rule 24  IF OC>=10.89 AND OC< 17.33  θ_S_ =0.676  Rule 25  IF OC>=17.33 AND OC<30.34  θ_S_ =0.771  Rule 26  IF OC>=30.34 AND OC< 48.63  θ_S_ =0.851  Rule 27  IF OC>=48.63  θ_S_ =0.914 |
| (3) | *θ*_S_  / cm^3^cm^-3^ | RT | Rule 1  IF USDA=S,SCL AND T/S=sub  θ_S_ =0.381  Rule 2  IF USDA=SL AND T/S=sub  θ_S_ =0.407  Rule 3  IF USDA=CL,L,LS,SiL AND T/S=sub  θ_S_ =0.428  Rule 4  IF USDA=CL,L,LS,S,SCL,SiL,SL AND T/S=top  θ_S_ =0.465  Rule 5  IF USDA=Si,SiC,SiCL  θ_S_ =0.470  Rule 6  IF USDA=C,SC  θ_S_ =0.520  Rule 7  IF USDA=O AND T/S=top  θ_S_ =0.767  Rule 8  IF USDA=O AND T/S=sub  θ_S_ =0.865 |
| (4) | *θ*_S_  / cm^3^cm^-3^ | RT | Rule 1  IF OC< 0.11 AND Sa>=37.37 AND Cl>=11.55  θ_S_ =0.328  Rule 2  IF OC< 0.11 AND Sa>=37.37 AND Cl< 11.55  θ_S_ =0.374  Rule 3  IF OC< 1.74 AND OC>=0.90 AND Sa>=37.37 AND T/S=sub  θ_S_ =0.375  Rule 4  IF OC< 0.90 AND OC>=0.11 AND Sa>=37.37 AND T/S=sub  θ_S_ =0.399  Rule 5  IF OC< 1.74 AND OC>=0.11 AND Sa>=37.37 AND T/S=top  θ_S_ =0.418  Rule 6  IF OC< 0.33 AND Sa< 37.37  θ_S_ =0.406  Rule 7  IF OC< 1.74 AND OC>=0.33 AND Sa< 37.37 AND Sa>=5.39 AND CL< 49.25  θ_S_ =0.451  Rule 8  IF OC< 1.74 AND OC>=0.33 AND Sa< 5.39 AND Cl< 49.25  THS=0.499  Rule 9  IF OC< 0.63 AND OC>=0.33 AND Sa< 37.37 AND Cl>=49.25  θ_S_ =0.463  Rule 10  IF OC< 1.74 AND OC>=0.63 AND Sa< 37.37 AND Cl>=49.25  θ_S_ =0.532  Rule 11  IF OC>=3.21 AND OC< 4.64 AND Cl< 52.15 AND Si< 59.03 AND Sa< 4.05  θ_S_ =0.382  Rule 12  IF OC>=1.74 AND OC< 3.21 AND Cl< 52.15 AND Si< 59.03 AND Sa< 4.05  θ_S_ =0.525  Rule 13  IF OC>=1.74 AND OC< 2.78 AND Cl< 52.15 AND Si< 59.03 AND Sa>=4.05  θ_S_ =0.461  Rule 14  IF OC>=2.78 AND OC< 4.64 AND Cl< 52.15 AND Si< 59.03 AND Sa>=4.05  θ_S_ =0.490  Rule 15  IF OC>=1.74 AND OC< 4.64 AND Cl< 52.15 AND Si>=59.03  θ_S_ =0.522  Rule 16  IF OC>=1.74 AND OC< 4.64 AND Cl>=52.15  THS=0.593  Rule 17  IF OC>=4.64 AND OC< 7.85 AND Sa>=29.51  θ_S_ =0.531  Rule 18  IF OC>=7.85 AND Sa>=29.51  θ_S_ =0.595  Rule 19  IF OC>=4.64 AND Sa< 29.51 AND T/S=top  θ_S_ =0.587  Rule 20  IF OC>=4.64 AND Sa< 29.51 AND T/S=sub  θ_S_ =0.695 |
| (5) | *θ*_S_  / cm^3^cm^-3^ | LRt | θ_S_ = 0.6819 - 0.06480 * (1/(OC+1)) - 0.11900 * BD^2^ - 0.02668 * T/S + 0.001489 * Cl + 0.0008031 * Si + 0.02321 * (1/(OC+1)) * BD^2^ + 0.01908 * BD^2^ * T/S - 0.0011090 * Cl * T/S - 0.00002315 * Si * Cl - 0.0001197 * Si * BD^2^ - 0.0001068 * Cl * BD^2^ |
| (6) | *θ*_S_  / cm^3^cm^-3^ | LRt | θ_S_ = 0.5653 - 0.07918 * BD^2^ + 0.001671 * pH^2^ + 0.0005438 * Cl + 0.001065 * Si + 0.06836 * T/S - 0.00001382 * Cl * pH^2^ - 0.00001270 * Si * Cl - 0.0004784 * BD^2^ * pH^2^ - 0.0002836 * Si * BD^2^ + 0.0004158 * Cl * BD^2^ - 0.01686 *T/S * BD^2^ - 0.0003541 * Si * T/S -0.0003152 * T/S * pH^2^ |
| (7) | *θ*_FC_  / cm^3^cm^-3^ | RT | Rule 1  IF FAO_MOD=coarse AND T/S=sub  θ_FC_ =0.157  Rule 2  IF FAO_MOD=coarse AND T/S=top  θ_FC_ =0.199  Rule 3  F FAO_MOD=medium AND T/S=sub  θ_FC_ =0.280  Rule 4  IF FAO_MOD=medium AND T/S=top  θ_FC_ =0.308  Rule 5  IF FAO_MOD=medium fine  θ_FC_ =0.326  Rule 6  IF FAO_MOD=fine, very fine  θ_FC_ =0.362  Rule 7  IF FAO_MOD=organic  θ_FC_ =0.575 |
| (8) | *θ*_FC_  / cm^3^cm^-3^ | RT | Rule 1  IF USDA=S  θ_FC_ =0.094  Rule 2  IF USDA=LS  θ_FC_ =0.165  Rule 3  IF USDA=Si,SL  θ_FC_ =0.236  Rule 4  IF USDA=SC,SCL AND T/S=sub  θ_FC_ =0.255  Rule 5  IF USDA=SC,SCL AND T/S=top  θ_FC_ =0.309  Rule 6  IF USDA=L,SiL  θ_FC_ =0.312  Rule 7  IF USDA=CL,SiCL AND T/S=sub  θ_FC_ =0.321  Rule 8  IF USDA=CL,SiCL AND T/S=top  θ_FC_ =0.355  Rule 9  IF USDA=C,SiC  θ_FC_ =0.373  Rule 10  IF USDA=O AND T/S=top  θ_FC_ =0.503  Rule 11  IF USDA=O AND T/S=sub  θ_FC_ =0.596 |
| (9) | *θ*_FC_  / cm^3^cm^-3^ | LRt | θ_FC_ = 0.2449 - 0.1887 * (1/(OC+1)) + 0.004527 * Cl + 0.001535 * Si + 0.001442 * Si * (1/(OC+1)) - 0.00005110 * Si * Cl + 0.0008676 * Cl * (1/(OC+1)) |
| (10) | *θ*_WP_  / cm^3^cm^-3^ | RT | Rule 1  IF FAO_MOD=coarse  θ_WP_ =0.069  Rule 2  IF FAO_MOD=medium  θ_WP_ =0.140  Rule 3  IF FAO_MOD=medium fine  θ_WP_ =0.163  Rule 4  IF FAO_MOD=fine, organic AND T/S=top  θ_WP_ =0.233  Rule 5  IF FAO_MOD=fine, organic AND T/S=sub  θ_WP_ =0.268  Rule 6  IF FAO_MOD=very fine  θ_WP_ =0.325 |
| (11) | *θ*_WP_  / cm^3^cm^-3^ | RT | Rule 1  IF USDA=LS,S,Si  θ_WP_ =0.050  Rule 2  IF USDA=SL  θ_WP_ =0.100  Rule 3  IF USDA=L,SiL  θ_WP_ =0.136  Rule 4  IF USDA=SCL  θ_WP_ =0.164  Rule 5  IF USDA=CL,SC,SiCL  θ_WP_ =0.211  Rule 6  IF USDA=C,O,SiC AND T/S=top  θ_WP_ =0.251  Rule 7  IF USDA=C,O,SiC AND T/S=sub  θ_WP_ =0.292 |
| (12) | *θ*_WP_  / cm^3^cm^-3^ | LRt | θ_WP_ = 0.09878 + 0.002127* Cl - 0.0008366 * Si - 0.07670 *(1/(OC+1)) + 0.00003853 * Si * Cl + 0.002330 * Cl * (1/(OC+1)) + 0.0009498 * Si * (1/(OC+1)) |
| (13) | log_10_(*K*_S_)  / log_10_(cm day^-1^) | RT | Rule 1  IF T/S=sub AND FAO_MOD=fine,very fine  log_10_(K_S_)= 0.01  Rule 2  IF T/S=sub AND FAO_MOD=coarse,medium,medium fine,organic  log_10_(K_S_)= 0.77  Rule 3  IF T/S=top AND FAO_MOD=coarse,fine,medium fine,organic,very fine  log_10_(K_S_)= 1.14  Rule 4  IF T/S=top AND FAO_MOD=medium  log_10_(K_S_)= 2.23 |
| (14) | log_10_(*K*_S_)  / log_10_(cm day^-1^) | RT | Rule 1  IF OC>=0.96 AND OC< 0.97 AND T/S=sub AND FAO_MOD=fine,medium,medium fine  log_10_(K_S_)= -1.16  Rule 2  IF OC>=0.96 AND OC< 0.97 AND T/S=sub AND FAO_MOD=coarse,very fine  log_10_(K_S_)= -0.44  Rule 3  IF OC>=1.52 AND OC< 1.54 AND T/S=sub  log_10_(K_S_)= -0.75  Rule 4  IF OC>=2.04 AND OC< 2.12 AND T/S=sub  log_10_(K_S_)= -0.58  Rule 5  IF OC>=2.65 AND OC< 3.86 AND T/S=sub  log_10_(K_S_)= -0.45  Rule 6  IF OC>=3.86 AND T/S=sub  log_10_(K_S_)= 0.37  Rule 7  IF OC>=2.12 AND OC< 2.65 AND T/S=sub  log_10_(K_S_)= 1.29  Rule 8  IF OC>=1.54 AND OC< 2.04 AND T/S=sub  log_10_(K_S_)= 1.33  Rule 9  IF OC>=0.97 AND OC< 1.52 AND T/S=sub  log_10_(K_S_)= 1.13  Rule 10  IF OC>=2.09 AND OC< 2.10 AND T/S=top  log_10_(K_S_)= -0.87  Rule 11  IF OC>=2.4 AND T/S=top AND FAO_MOD=fine,medium fine,organic,very fine  log_10_(K_S_)= -0.38  Rule 12  IF OC>=2.10 AND OC< 2.40 AND T/S=top AND FAO_MOD=fine,medium fine,organic,very fine  log_10_(K_S_)= 1.67  Rule 13  IF OC>=2.10 AND T/S=top AND FAO_MOD=coarse,medium  log_10_(K_S_)= 1.19  Rule 14  IF OC>=1.52 AND OC< 1.54 AND T/S=top  log_10_(K_S_)= -0.49  Rule 15  IF OC< 2.09 AND OC>=1.54 AND T/S=top  log_10_(K_S_)= 1.74  Rule 16  IF OC>=0.96 AND OC< 1.52 AND T/S=top  log_10_(K_S_)= 1.80  Rule 17  IF OC< 0.41 AND OC>=0.40 AND FAO_MOD=fine,medium,medium fine,very fine  log_10_(K_S_)= -1.56  Rule 18  IF OC>=0.40 AND OC< 0.41 AND FAO_MOD=coarse  log_10_(K_S_)= -0.42  Rule 19  IF OC< 0.96 AND OC>=0.41 AND FAO_MOD=fine,very fine  log_10_(K_S_)= 0.79  Rule 20  IF OC< 0.96 AND OC>=0.41 AND FAO_MOD=coarse,medium,medium fine  log_10_(K_S_)= 1.54  Rule 21  IF OC< 0.07  log_10_(K_S_)= 0.55  Rule 22  IF OC< 0.40 AND OC>=0.07 AND FAO_MOD=fine,very fine  log_10_(K_S_)= 0.66  Rule 23  IF OC< 0.40 AND OC>=0.07 AND FAO_MOD=medium,medium fine  log_10_(K_S_)= 1.30  Rule 24  IF OC< 0.40 AND OC>=0.07 AND FAO_MOD=coarse  log_10_(K_S_)= 1.83 |
| (15) | log_10_(*K*_S_)  / log_10_(cm day^-1^) | RT | Rule 1  IF USDA=C,O,SC,SCL,Si,SiC,SiCL  log_10_(K_S_)= 0.12  Rule 2  IF USDA=CL,L,LS,S,SiL,SL AND T/S=sub  log_10_(K_S_)= 0.82  Rule 3  IF USDA=LS,S,SiL,SL AND T/S=top  log_10_(K_S_)= 1.47  Rule 4  IF USDA=CL,L AND T/S=top  log_10_(K_S_)= 2.69 |
| (16) | log_10_(*K*_S_)  / log_10_(cm day^-1^) | RT | Rule 1  IF OC>=0.96 AND OC< 0.97 AND T/S=sub  log_10_(K_S_)= -0.95  Rule 2  IF OC>=1.52 AND OC< 1.54 AND T/S=sub  log_10_(K_S_)= -0.75  Rule 3  IF OC>=2.04 AND T/S=sub  log_10_(K_S_)= -0.25  Rule 4  IF OC>=1.54 AND OC< 2.04 AND T/S=sub  log_10_(K_S_)= 1.33  Rule 5  IF OC>=0.97 AND OC< 1.52 AND T/S=sub  log_10_(K_S_)= 1.13  Rule 6  IF OC>=2.09 AND OC< 2.10 AND T/S=top  log_10_(K_S_)= -0.87  Rule 7  IF OC>=2.42 AND T/S=top AND Sa< 38.95  log_10_(K_S_)= -0.22  Rule 8  IF OC>=2.10 AND OC< 2.42 AND T/S=top AND Sa< 38.95  log_10_(K_S_)= 1.82  Rule 9  IF OC>=2.10 AND T/S=top AND Sa>=38.95  log_10_(K_S_)= 1.44  Rule 10  IF OC>=0.96 AND OC< 2.09 AND T/S=top AND Si< 10.85  log_10_(K_S_)= 0.01  Rule 11  IF OC>=1.52 AND OC< 1.54 AND T/S=top AND Si>=10.85  log_10_(K_S_)= -0.46  Rule 12  IF OC>=1.54 AND OC< 2.09 AND T/S=top AND Si>=10.85  log_10_(K_S_)= 1.72  Rule 13  IF OC>=0.96 AND OC< 1.52 AND T/S=top AND Si>=10.85  log_10_(K_S_)= 1.82  Rule 14  IF OC< 0.41 AND OC>=0.40 AND Si>=32.11  log_10_(K_S_)= -1.81  Rule 15  IF OC>=0.40 AND OC< 0.41 AND Si< 32.11  log_10_(K_S_)= -0.40  Rule 16  IF OC< 0.96 AND OC>=0.41 AND Cl>=37.4  log_10_(K_S_)= 0.67  Rule 17  IF OC< 0.96 AND OC>=0.41 AND Cl< 37.4  log_10_(K_S_)= 1.53  Rule 18  IF OC< 0.07  log_10_(K_S_)= 0.55  Rule 19  IF OC< 0.40 AND OC>=0.07 AND Sa< 5.77  log_10_(K_S_)= -0.11  Rule 20  IF OC< 0.40 AND OC>=0.07 AND Sa< 69.72 AND Sa>=5.77  log_10_(K_S_)= 1.28  Rule 21  IF OC< 0.40 AND OC>=0.07 AND Sa>=69.72  log_10_(K_S_)= 1.96 |
| (17) | log_10_(*K*_S_)  / log_10_(cm day^-1^) | LR | log_10_K_S_ = 0.40220 + 0.26122 * pH + 0.44565 * T/S - 0.02329 * Cl - 0.01265 * Si - 0.01038 * CEC |
| (18) | MRC and HCC (*θ*_r_, *θ*_s_, *α*, *n, K*_0_*, L* parameters of MVG model) | MS | \| c \| Modified FAO texture classes \| MVG parameters \| \| \| \| \| \| \| \| --- \| --- \| --- \| --- \| --- \| --- \| --- \| --- \| --- \| \| *θ*_r_  (cm^3^ cm^-3^) \| *θ*_s_  (cm^3^ cm^-3^) \| *α*  (cm^-1^) \| *n*  (-) \| *m*  (-) \| *K*_0_  (cm day^-1^) \| *L*  (-) \| \| Top-soils \| coarse \| 0.045 \| 0.438 \| 0.0478 \| 1.3447 \| 0.2563 \| 17.30 \| -2.5587 \| \| medium \| 0.000 \| 0.459 \| 0.0309 \| 1.1920 \| 0.1611 \| 12.49 \| -3.8570 \| \| medium fine \| 0.000 \| 0.432 \| 0.0094 \| 1.2119 \| 0.1749 \| 1.68 \| -4.4460 \| \| fine \| 0.000 \| 0.478 \| 0.0403 \| 1.1176 \| 0.1053 \| 40.19 \| -4.7040 \| \| very fine \| 0.000 \| 0.522 \| 0.0112 \| 1.1433 \| 0.1253 \| 2.69 \| -5.0000 \| \| organic \| 0.111 \| 0.697 \| 0.0069 \| 1.4688 \| 0.3192 \| 1.42 \| 0.3284 \| \| Sub-soils \| coarse \| 0.057 \| 0.404 \| 0.0426 \| 1.5349 \| 0.3485 \| 9.68 \| -1.8191 \| \| medium \| 0.000 \| 0.428 \| 0.0347 \| 1.1725 \| 0.1471 \| 11.78 \| -4.9869 \| \| medium fine \| 0.000 \| 0.418 \| 0.0066 \| 1.2173 \| 0.1785 \| 1.87 \| -3.3761 \| \| fine \| 0.000 \| 0.430 \| 0.0011 \| 1.2290 \| 0.1863 \| 0.07 \| -1.8486 \| \| very fine \| 0.000 \| 0.511 \| 0.0002 \| 1.4048 \| 0.2882 \| 0.02 \| 5.0000 \| \| organic \| 0.000 \| 0.835 \| 0.0113 \| 1.2256 \| 0.1841 \| 10.81 \| 2.7337 \| |
| (19) | MRC and HCC (*θ*_r_, *θ*_s_, *α*, *n, K*_0_*, L* parameters of MVG model) | MS | \|  \| USDA texture classes \| MVG parameters \| \| \| \| \| \| \| \| --- \| --- \| --- \| --- \| --- \| --- \| --- \| --- \| --- \| \| *θ*_r_  (cm^3^ cm^-3^) \| *θ*_s_  (cm^3^ cm^-3^) \| *α*  (cm^-1^) \| *n*  (-) \| *m*  (-) \| *K*_0_  (cm day^-1^) \| *L*  (-) \| \| Top-soils \| sand \| 0.061 \| 0.411 \| 0.0258 \| 1.8005 \| 0.4446 \| 8.33 \| -0.7306 \| \| loamy sand \| 0.052 \| 0.475 \| 0.0341 \| 1.4846 \| 0.3264 \| 8.95 \| -1.8749 \| \| sandy loam \| 0.000 \| 0.441 \| 0.0750 \| 1.1904 \| 0.1599 \| 44.88 \| -4.3523 \| \| loam \| 0.000 \| 0.491 \| 0.0347 \| 1.1931 \| 0.1618 \| 14.17 \| -4.3000 \| \| silt loam \| 0.000 \| 0.424 \| 0.0074 \| 1.2545 \| 0.2029 \| 1.17 \| -3.5496 \| \| silt \| 0.009 \| 0.465 \| 0.0042 \| 1.4853 \| 0.3267 \| 1.38 \| -2.6418 \| \| sandy clay loam \| 0.000 \| 0.409 \| 0.0700 \| 1.1335 \| 0.1178 \| 43.63 \| -5.0000 \| \| clay loam \| 0.000 \| 0.465 \| 0.1284 \| 1.1160 \| 0.1040 \| 195.15 \| -5.0000 \| \| silty clay loam \| 0.000 \| 0.463 \| 0.0107 \| 1.1892 \| 0.1591 \| 1.38 \| -2.6418 \| \| sandy clay \| 0.192 \| 0.523 \| 0.0351 \| 1.4455 \| 0.3082 \| 43.80 \| -1.6202 \| \| silty clay \| 0.000 \| 0.455 \| 0.0309 \| 1.1110 \| 0.0999 \| 0.01 \| 5.0000 \| \| clay \| 0.000 \| 0.499 \| 0.0234 \| 1.1200 \| 0.1072 \| 17.07 \| -5.0000 \| \| organic \| 0.111 \| 0.697 \| 0.0069 \| 1.4688 \| 0.3192 \| 1.42 \| 0.3284 \| \| Sub-soils \| sand \| 0.034 \| 0.368 \| 0.0356 \| 1.7767 \| 0.4372 \| 5.97 \| -1.4096 \| \| loamy sand \| 0.037 \| 0.423 \| 0.0419 \| 1.4222 \| 0.2968 \| 14.84 \| -1.9583 \| \| sandy loam \| 0.000 \| 0.437 \| 0.0681 \| 1.1966 \| 0.1643 \| 53.50 \| -3.7279 \| \| loam \| 0.000 \| 0.432 \| 0.0336 \| 1.1701 \| 0.1454 \| 8.58 \| -5.0000 \| \| silt loam \| 0.000 \| 0.422 \| 0.0077 \| 1.2483 \| 0.1989 \| 1.76 \| -3.3247 \| \| silt \| 0.009 \| 0.465 \| 0.0042 \| 1.4853 \| 0.3267 \| 0.45 \| -5.0000 \| \| sandy clay loam \| 0.000 \| 0.384 \| 0.0717 \| 1.1206 \| 0.1076 \| 37.09 \| -5.0000 \| \| clay loam \| 0.000 \| 0.413 \| 0.0227 \| 1.1191 \| 0.1064 \| 12.35 \| -5.0000 \| \| silty clay loam \| 0.000 \| 0.408 \| 0.0032 \| 1.1993 \| 0.1662 \| 0.45 \| -5.0000 \| \| sandy clay \| 0.000 \| 0.365 \| 0.0016 \| 1.1812 \| 0.1534 \| 43.80 \| -1.6202 \| \| silty clay \| 0.000 \| 0.442 \| 0.0003 \| 1.3861 \| 0.2786 \| 0.01 \| 5.0000 \| \| clay \| 0.000 \| 0.461 \| 0.0004 \| 1.3027 \| 0.2323 \| 0.04 \| 1.1840 \| \| organic \| 0.000 \| 0.835 \| 0.0113 \| 1.2256 \| 0.1841 \| 10.81 \| 2.7337 \| |
| (20) | MRC (*θ_r_* / cm^3^ cm^-3^, *θ_s_* / cm^3^ cm^-3^, log_10_(*α*) / log_10_(cm^-1^), log_10_(*n*-1) / - parameters of VG model) | RT (for *θ_r_*) and LRt (for *θ_s_*, log_10_(*α*) and log_10_(*α*)) | \| Rule 1  IF Sa >= 2.00  θ_r_ = 0.041  Rule 2  IF Sa < 2.00  θ_r_ = 0.179 \| \| --- \| \| θ_s_ = 0.5056 - 0.1437 * (1/(OC+1)) + 0.0004152 * Si \| \| log_10_(α) = -1.3050 - 0.0006123 * Si - 0.009810 * Cl + 0.07611 * (1/(OC+1)) - 0.0004508 * Si * Cl + 0.03472 * Cl * (1/(OC+1)) - 0.01226 * Si * (1/(OC+1))  log_10_(n-1) = 0.01516 - 0.005775 * (1/(OC+1)) - 0.24885 * log_10_(CEC) - 0.01918 * Cl - 0.0005052 * Si - 0.007544 * pH^2^ - 0.02159 * Cl * (1/(OC+1)) + 0.01556 * Cl * log_10_(CEC) + 0.01477 * (1/(OC+1)) * pH^2^ + 0.0001121 * Si * Cl - 0.33198 * (1/(OC+1)) * log_10_(CEC) \| |
| (21) | MRC (*θ_r_* / cm^3^ cm^-3^, *θ_s_* / cm^3^ cm^-3^, log_10_(*α*) / log_10_(cm^-1^), log_10_(*n*-1) / - parameters of VG model) | RT (for *θ_r_*) and LR (for *θ_s_*, log_10_(*α*) and log_10_(*α*)) | \| Rule 1  IF Sa >= 2.00  θ_r_ = 0.041  Rule 2  IF Sa < 2.00  θ_r_ = 0.179 \| \| --- \| \| θ_s_ = 0.83080 - 0.28217 * BD + 0.0002728 * Cl + 0.000187 * Si \| \| log_10_(α) = -0.43348 - 0.41729 * BD - 0.04762 * OC + 0.21810 * T/S - 0.01581 * Cl - 0.01207 * Si \| \| log_10_(n-1) = 0.22236 - 0.30189 * BD -0.05558 * T/S - 0.005306 * Cl - 0.003084 * Si - 0.01072 * OC \| |
| (22) | MRC (*θ_r_* (cm^3^ cm^-3^), *θ_s_* (cm^3^ cm^-3^), log_10_(*α*) (log_10_(cm^-1^)) , log_10_(*n*-1) (-) parameters of VG model) | RT (for *θ_r_*) and LRt2 (for *θ_s_*, log_10_(*α*) and log_10_(*α*)) | \| Rule 1  IF Sa >= 2.00  θ_r_ = 0.041  Rule 2  IF Sa < 2.00  θ_r_ = 0.179 \| \| --- \| \| θ_s_ = 0.63052 - 0.10262 * BD^2^ + 0.0002904 * pH^2^ + 0.0003335 * Cl \| \| log_10_(α) = -1.16518 + 0.40515 * (1/(OC+1)) - 0.16063 * BD^2^ - 0.008372 * Cl - 0.01300* Si + 0.002166 * pH^2^ + 0.08233 * T/S \| \| log_10_(n-1) = -0.25929 + 0.25680 * (1/(OC+1)) - 0.10590 * BD^2^ - 0.009004 * Cl - 0.001223 * Si \| |

^a^Abbreviations used in table: FAO_MOD: modified FAO texture classes (5 class for mineral soils and an organic class); USDA: USDA-SCS texture classes and an organic class (S: sand, LS: loamy sand, SL: sandy loam, L: loam, SiL: silt loam, Si: silt, SCL: sandy clay loam, CL: clay loam, SiCL: silty clay loam, SC: sandy clay, SiC: silty clay, C: clay, O: organic soils); Sa: sand content (50-2000 µm) (%); Si: silt content (2-50 µm) (%); Cl: clay content (0-2 µm) (%); T/S: top-soil (top) and sub-soil (sub) distinction; OC: organic carbon content (%); BD: bulk density (g cm^-3^); pH: pH in water; CEC: cation exchange capacity (meq 100g^-1^); *θ*_S_: saturated water content (cm^3^ cm^-3^); *θ*_FC_: water content at field capacity (cm^3^ cm^-3^); *θ*_WP_: water content at wilting point (cm^3^ cm^-3^); *K*_S_: saturated hydraulic conductivity log_10_(cm day^-1^)]; MRC: moisture retention curve; HCC: hydraulic conductivity curve; VG: van Genuchten model; MVG: Mualem-van Genuchten model; MS: mean statistics of developer determined groups; RT: univariate regression tree; LR, LRt, LRt2: linear regression with or without transformation and interactions, further details of the LR models are provided in ‘Basic principles for fitting the linear regression (LR) model’ section.

**References**

Batjes, N. H. 2009. Harmonized soil proﬁle data for applications at global and continental scales: updates to the WISE database. *Soil Use & Management*, **25**, 124–127.)

Breiman, L., Friedman, J.H., Stone, C.J. & Olshen, R.A. 1984. *Classification and Regression Trees.* Chapman & Hall/CRC, New York, USA.

Commission of the European Communities, CEC. 1985. Soil map of the European Communities. Scale 1:1000000. CEC-DGVI, Luxembourg, Luxemburg..

De’ath, G. 2002. Multivariate Regression Trees : A new technique for modeling species-environment relationships. *Ecology*, **83**, 1105–1117.

IPCC (Intergovernmental Panel on Climate Change) 2003. Penman J., M. Gytarsky, T. Hiraishi, T. Krug, D. Kruger, R. Pipatti, *et al.* (eds). *Good Practice Guidance for Land Use, Land Use Change and Forestry.* IPCC/OECD/IEA/IGES, Hayama, Japan

Ippisch, O., Vogel, H.J. & Bastian, P. 2006. Validity limits for the van Genuchten-Mualem model and implications for parameter estimation and numerical simulation. *Advances in Water Resources,* **29**, 1780–1789.

McKenzie, N. & Jacquier, D. 1997. Improving the field estimation of saturated hydraulic conductivity in soil survey. *Australian Journal of Soil Research,* **35**, 803–827.

Richards, L.A. 1931. Capillary conduction of liquids through porous mediums. *Physics,* **1**, 318–333.

Schaap, M.G. & van Genuchten, M.T. 2006. A modified Mualem-van Genuchten formulation for improved description of the hydraulic conductivity near saturation. *Vadose Zone Journal,* **5**, 27.
